# Exploring the BICC1 Interactome in HEK293T Cells: Insights Into RNA‐Mediated Protein Networks and Biomolecular Condensates

**DOI:** 10.1096/fba.2026-00035

**Published:** 2026-08-07

**Authors:** Heloísa Monteiro do Amaral‐Prado, Gabriela Alves Moreira, Cristiane Santos, Fernando Henrique Bosso, Felipe Eduardo Ciamponi, Guilherme Oliveira Barbosa, Mario Henrique Bengtson, Katlin Brauer Massirer

**Affiliations:** ^1^ Center for Medicinal Chemistry ‐ CQMED, Center for Molecular Biology and Genetic Engineering ‐ CBMEG Universidade Estadual de Campinas Campinas São Paulo Brazil; ^2^ Department of Biochemistry and Tissue Biology, Institute of Biology Universidade Estadual de Campinas São Paulo Brazil

**Keywords:** membraneless organelles, PRMT5 methylosome, RNA‐binding proteins, splicing, stress granules

## Abstract

Bicaudal C Homolog 1 (BICC1) is a conserved RNA‐binding protein that, in mammals, has been primarily associated with polycystic kidney disease and renal organogenesis. However, its role in other disease contexts, including cancer, remains poorly understood. In this study, we characterized the BICC1 interactome, with emphasis on its dependence on RNA and the sterile alpha motif (SAM) domain, to identify novel biological processes associated with BICC1 function. Protein complexes were purified from HEK293T cells by co‐immunoprecipitation and analyzed by mass spectrometry. Notably, co‐immunoprecipitations performed in the presence of RNA yielded a larger number of interacting proteins, with 31 of 71 proteins (~43%) uniquely identified under RNA‐preserved conditions, highlighting the critical role of RNA in mediating BICC1 protein–protein interactions. Enriched proteins were predominantly associated with mRNA splicing, the PRMT5 methylosome complex, and membraneless organelles, such as biomolecular condensates. Consistent with these findings, immunofluorescence assays performed on stressed cells revealed the co‐localization of BICC1 with stress granule markers. Moreover, BICC1 interactions with PRMT5, STK38, PARP1, and IGF2BP1 were confirmed by immunoblotting.

## Graphical abstract

Workflow for BICC1 interactome characterization in HEK293T cells. Cells expressing FLAG‐tagged BICC1 (full‐length or ΔSAM) were subjected to co‐immunoprecipitation under RNA‐preserved (−) or RNase‐treated (+) conditions (1). Interacting proteins were identified by LC–MS/MS (2) and analyzed through protein–protein interaction networks and functional enrichment (3). Key partners were validated by immunoblotting and immunofluorescence, revealing RNA‐dependent interactions and associations with mRNA splicing, the PRMT5 methylosome, and membraneless organelles, such as stress granules (4).

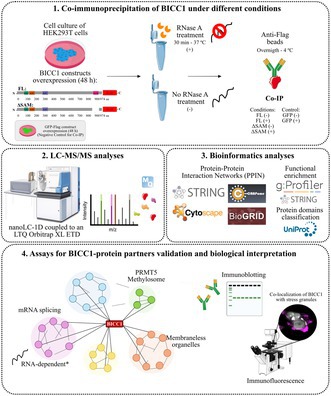

**Abbreviations**
(+): Treatment with RNase A
(−): No treatment with RNase A
ABC: Ammonium Bicarbonate Buffer
ACN: Acetonitrile
BICC1: Bicaudal C Homolog 1
CCT2: T‐complex protein 1 subunit beta
Co‐IP‐MS: Co‐immunoprecipitation followed by mass spectrometry
CSD: Cold Shock Domain
DAPI: 4′,6‐diamidino‐2‐phenylindole
DDA: Data dependent analysis
DDX: DEAD‐box RNA helicases
DTT: Dithiothreitol
FA: Formic acid
FL: Construct of the full‐length sequence of BICC1 in pcDNA.3.1 vector backbone
GFP: Green fluorescent protein
GO: Gene ontology
HEK293T: Human embryonic kidney cell line
IF: Immunofluorescence
IGF2BP1: Insulin‐like growth factor 2 mRNA‐binding protein 1
KH: K‐homology domain
LC: Liquid chromatography
LLPS: Liquid–liquid phase separation
MLO: Membraneless organelles
MS: Mass spectrometry
PARP1: Poly [ADP‐ribose] polymerase 1
P‐body: RNA‐processing bodies
PC: Pearson's correlation
PFA: Paraformaldehyde
PKD: Polycystic kidney disease
PPIN: Protein–protein interaction network
PRMT5: Protein arginine methyltransferase 5
RBD: RNA‐binding domain
RBP: RNA‐binding protein
RRM: RNA‐recognition motif
RT: Room temperature
SAM: Sterile alpha motif domain
SGs: Stress granules
STK38: Serine/threonine kinase 38
WDR77: Methylosome protein WDR77
Wnt: Wingless‐related integration site
ΔSAM: Deletion of the sterile alpha motif domain

## 1 Introduction

RNA‐binding proteins (RBPs) play a crucial role in determining the RNA fate and function within the cell through binding to single‐ or double‐stranded RNA and assembling into ribonucleoprotein complexes (RNPs), whose composition varies according to the cellular context [1]. Through these interactions, RBPs mediate posttranscriptional regulation of RNA, including splicing, intracellular trafficking, translation, stabilization, and RNA degradation in the cytoplasm [1].

The Bicaudal C Homolog 1 (BICC1) is a conserved RBP found across multiple organisms (Figure S1). It was originally identified through mutations at a locus in *Drosophila melanogaster* that caused the formation of duplicated posterior structures during embryonic development, giving rise to the name *Bicaudal* [2, 3, 4]. Structurally, BICC1 contains three K‐Homology domains (KH) and two KH‐like domains (KHL1 and KHL2) at its N‐terminus. These domains harbor the conserved “GXXG” motifs characteristic of RBPs [5, 6]. The C‐terminal region of BICC1 contains a sterile alpha motif domain (SAM), which is implicated in protein–protein interactions, self‐association, and, in some contexts, RNA binding activities [7].

In mammals, the studies about BICC1 started when mutations in this RBP were related to the development of the polycystic kidney disease (PKD) and renal organogenesis in mice and human patients [8, 9, 10]. Mechanistically, BICC1 is mostly identified as a negative regulator of selected mRNAs and signaling pathways, as demonstrated in the Wnt/β‐catenin signaling [11]. In both cases, the SAM or KH domains of this protein play a crucial role in its regulation [10, 11, 12]. Several transcripts are targeted by BICC1, which are critically involved in diverse biological activities, including development and cell differentiation [13], and metabolism [14]. A gradient of maternal Bicaudal‐C has been shown to regulate embryogenesis in vertebrate models, such as *Xenopus laevis* , through translational repression of mRNAs encoding regulators of cell fate [15], and to modulate metabolic processes in murine models, including gluconeogenesis, which are linked to cyst growth and proliferation‐associated pathways [16]. Moreover, an increase in the expression of BICC1 has been demonstrated to correlate with various types of cancer, such as glioblastoma [17], gastric [18], oral [19], and pancreatic cancer [14, 20], and neurological disorders [21]. However, these are still exploratory works, with no mechanistic insights well described yet.

Exploring the interactome of RBPs, such as BICC1, under RNA‐dependent conditions is an effective strategy to advance our understanding of their cellular function, particularly when their roles in disease contexts remain understudied. In interactome databases, including BioGRID [22], most of the proteins identified as BICC1 partners derive from high‐throughput studies. To date, BICC1 has been well characterized as a binding partner of ANKS3 and ANKS6, as demonstrated by co‐immunoprecipitation in different cell lines [8, 23, 24], and these interactions are thought to play a role in ciliary signaling pathways [8]. In addition, BICC1 has been identified as a binding partner of the CCR4–NOT deadenylase complex through a direct interaction with its Not3/5 subunit, initially in *Drosophila melanogaster* , and more recently in mammalian cells [13, 25]. In both cases, this interaction is critical for mRNA degradation. However, it remains unclear whether the BICC1 interactome is modulated by the presence of mRNAs or by specific structural domains of the protein.

Here, we expanded the characterization of the BICC1 interactome in HEK293T cells by co‐immunoprecipitation of Flag‐tagged BICC1 constructs followed by mass spectrometry (Co‐IP‐MS), assessing the dependence of these interactions on RNA and on the SAM domain. In total, 71 proteins were identified as the most probable BICC1 interaction partners, and a subset of these interactions was validated by immunoblotting and immunofluorescence assays. Protein–protein interaction network (PPIN) analysis and Gene Ontology (GO) annotation revealed significant enrichment of biological processes related to mRNA processing and splicing, involving the spliceosome, the methylosome complex, and membraneless organelles such as stress granules (SGs), P‐bodies, and Cajal bodies. Collectively, this approach substantially advances the functional characterization of BICC1, providing new insights into its interactome and its role in cellular biology.

## 2 Materials and Methods

### 2.1 Cell Culture and Transfection

The HEK293T human kidney embryonic cell line (CVCL_0045) was obtained from the American Type Culture Collection (ATCC). The cells were cultured in Dulbecco's Modified Eagle's Media (São Paulo, Brazil, Thermo Scientific, #11965092) supplemented with 10% fetal bovine serum (São Paulo, Brazil, Cultilab, #F063) and 1% penicillin–streptomycin (São Paulo, Brazil, Sigma, #P4333). Cells were grown in an incubator with 5% CO_2_ at 37°C. Subculturing was performed using 0.25% trypsin–EDTA (Thermo Scientific, #25200–072) with media replacement every two days. For transfection of plasmid DNA, Lipofectamine 3000 transfection reagent (Thermo Scientific, #L3000001) was employed according to the manufacturer's protocol. The standard transfection duration was 48 h.

### 2.2 Cloning and Plasmid Constructs

The vector pcDNA3.1 containing the corresponding sequence of Flag‐tagged BICC1 (Full‐Length sequence of BICC1—FL) was obtained from our collaborator, Prof. Dr. Opher Gilead, at the Structural Genomics Consortium (SGC) in Oxford. This plasmid was constructed by inserting the human BICC1 coding sequence, CDS, (NM_001080512.3) in frame with the Flag tag at the C‐terminal position. To create a variant lacking the SAM domain (designated as ΔSAM) of BICC1, we employed site‐directed mutagenesis, using the following specific primers:

*F 5′‐gctgtaacttaaatagctctcgaagaaagctttttgaatc‐3′*;

*R 5′‐gattcaaaaagctttcttcgagagctatttaagttacag‐3′*.

The construct of pcDNA3‐GFP‐Flag was used as a transfection control and as a negative control for Flag tag co‐immunoprecipitation. The STK38 construct fused to NanoLuc luciferase (STK38‐NanoLuc) was kindly donated by Promega Corporation (São Paulo, Brazil, #NV2121).

### 2.3 Silver Nitrate Staining of Proteins in SDS‐PAGE

For gel staining with silver nitrate, the gels were first fixed with a fixative solution (50% methanol, 12% acetic acid, and 0.2% formaldehyde) for 30 s in the microwave (maximum power) and incubated for 5 min at room temperature (RT) in a shaker. Then the gels were incubated with a solution of 30% ethanol for 30 s in the microwave (maximum power) and incubated for 5 min at RT in a shaker and after that, the gels were treated with a solution of reducing agent sodium thiosulphate (1.265 mM sodium thiosulphate) for 30 s in the microwave (maximum power) and incubated for 2 min at RT in a shaker. Gels were then washed twice with distilled water for 30 s in the microwave (maximum power) and incubated for 2 min at RT in a shaker, immediately after they were submitted to silver nitrate treatment protected from light (11 mM silver nitrate and 0.075% formaldehyde) for 30 s in the microwave (maximum power) and incubated for 5 min at RT in a shaker. Then the gels were washed with distilled water for 20 s at RT in a shaker and incubated with a revealing solution (599 mM calcium carbonate, 0.05% formaldehyde, and 25 μM sodium thiosulphate). The stop solution (50% methanol and 12% acetic acid) was added immediately after the appearance of the bands at the desired intensity. Silver staining was used as a qualitative method to assess protein enrichment and overall banding patterns; no quantitative analysis was performed.

### 2.4 Co‐Immunoprecipitation (Co‐IP)

Co‐immunoprecipitation (Co‐IP) approach was utilized to isolate BICC1 and its associated protein partners. Cells were seeded in 100 mm plates at a confluence of 70%–90% for each experimental condition and performed in technical triplicate. Following transfection, proteins were extracted using lysis buffer (50 mM Tris–HCl pH 7.4, 1 mM EDTA, 150 mM NaCl, 0.5% Triton X‐100, 10% Glycerol, 1 mM PMSF, all in DEPC‐treated water). Additionally, a protease inhibitor cocktail was included in the lysis buffer to enhance protein stability during extraction. Protein concentration was determined through the BCA assay (Pierce BCA Protein Assay Kit, Thermo Scientific, #23225), and 1 mg of cell lysate was incubated with 20 μL of anti‐flag M2 magnetic beads (Sigma, #M8823) for immunoprecipitation overnight at 4°C. The resulting eluates were set aside for either mass spectrometry analysis or immunoblotting. In the RNA preservation conditions, 150 U of Recombinant Ribonuclease Inhibitor (RNaseOUT, Thermo Scientific, #10777–019) was employed, while for RNA‐protein complex disruption conditions, 15 μg of recombinant RNase A was used (São Paulo, Brazil, Merckerey‐Nagel, #740505) for 30 min at 37°C.

### 2.5 SDS‐PAGE and Immunoblotting

Proteins from Co‐IP were eluted from anti‐Flag M2 magnetic beads through incubation with 20 μL of SDS‐PAGE (SDS polyacrylamide gel electrophoresis) loading buffer (Tris–HCl pH 6.8 40 mM, SDS 1%, β‐mercaptoethanol (14.7 M) 2,5%, Glycerol 6%, Bromophenol blue 0.005%) at 95°C for 10 min. For immunoblotting, proteins were separated by SDS‐PAGE and transferred onto polyvinylidene fluoride (PVDF) membranes using a semi‐dry transfer system (BIO‐RAD, Trans‐Blot Turbo). Membranes were first blocked with 5% bovine serum albumin (BSA) in tris‐buffered saline with 0.1% Tween 20 detergent (TBS‐t) for 30 min at RT, followed by incubation with primary antibodies and secondary antibodies, according to Table S1 conditions, both diluted in TBS‐t with 2% BSA. After each incubation with primary or secondary antibodies, the membranes were washed three times with TBS‐t for 5 min at RT. The blots were obtained by fluorescence exposure (Li‐cor Odyssey CLX scanner).

### 2.6 In Gel Digestion With Trypsin

Co‐immunoprecipitated protein samples were subjected to a short SDS‐PAGE run, allowing proteins to migrate only into the top portion of the resolving gel. The corresponding gel region was then excised and processed for in gel digestion. Briefly, protein‐containing gel regions were excised and subjected to the following protocol: Gel pieces were dehydrated with 100% ethanol, and mixed in the thermomixer for 10 min at 25°C (800 rpm). Next, the supernatant was removed, and the samples were dried in a speed‐vac for 7 min (vacuum pressure = 0.1). The samples were subjected to a reduction step by using a 10 mmol/L DTT solution in ABC (Ammonium Bicarbonate Buffer) at 50 mmol/L for 60 min at 56°C (800 rpm). Next, they were alkylated with 55 mmol/L iodoacetamide solution in ABC at 50 mmol/L for 45 min at 25°C (800 rpm) while being protected from light. Following this step, the gel pieces were subjected again to dehydration. Finally, 12.5 ng/μL of diluted trypsin solution was added to the dried gel pieces with 50 mmol/L ABC solution for 20 min at 4°C. Subsequently, the samples were incubated at 37°C for 16–18 h and the peptides extraction was performed using extraction solution with 3% TFA (trifluoroacetic acid) and 30% ACN (acetonitrile) in thermomixer for 10 min at 25°C (800 rpm), twice. ACN was added for 10 min at 25°C (800 rpm) twice and dried in a speed‐vac to 10%–20% of the original volume to remove ACN. Finally, the dried peptides were stored at −20°C for posterior desalting and purification.

### 2.7 Desalinization and Peptides Purification

Peptides were desalted and purified using C18 StageTips (São Paulo, Brazil, 3 M EmporeT) preconditioned with 100% methanol and equilibrated with 0.1% FA (formic acid). Peptides were eluted in a refrigerator using 20 μL of 40% ACN in 0.1% FA, applied twice (final volume: 40 μL). The eluates were dried in a Speed‐vac for 30 min without heating (vacuum pressure = 0.1) to remove ACN. Just before injection into the mass spectrometer, samples were resuspended in 31 μL in 0.1% FA with 5% DMSO and 5% ACN. Peptide concentration was determined using a NanoDrop spectrophotometer, and 5 μL (total mass of 135 ng) was injected into the LC–MS/MS system.

### 2.8 Liquid Chromatography and Mass Spectrometry Analyses (LC–MS/MS)

Samples from each experimental condition were analyzed in technical triplicates using a nanoLC‐1D Plus system (Eksigent) coupled to an LTQ Orbitrap XL ETD mass spectrometer (Thermo Scientific). Peptides were separated on an analytical PicoFrit column (15 cm × 75 μm ID, 3 μm C18 particle size) using a linear gradient of 2%–40% ACN in 0.1% FA, at a flow rate of 250 nL/min over 60 min. The nanospray ion source was operated at a capillary temperature of 175°C, and internal mass calibration was performed using a lock mass of m/z 401.92. The mass spectrometer was operated in DDA (data‐dependent acquisition) mode, with full MS scans acquired over the m/z range of 300–1800. Ion injection targets were set to 1 × 10^6^ for full MS scans and 3 × 10^5^ for MS/MS scans. The ten most intense precursor ions from each scan were selected for collision‐induced dissociation, with dynamic exclusion enabled for 90 s. Files were processed using MaxQuant software (version 2.2.0.0), incorporating the Andromeda algorithm. Spectra were searched against the UniProt human reference proteome database using the following parameters: precursor mass tolerance of 0.5 Da, fragment mass tolerance of 0.15 Da, carbamidomethylation of cysteine (C) as a fixed modification, and oxidation (M), acetylation (protein N‐terminus), and methylation (E, K, R) as variable modifications. The FDR (false discovery rate) was set to 1% at both the protein and peptide‐spectrum match levels.

### 2.9 Bioinformatics

Proteins immunoprecipitated with both Flag‐tagged BICC1 variants (FL and ΔSAM) and identified by mass spectrometry were analyzed using a bioinformatics workflow to identify enriched biological processes and cellular components within the BICC1 interactome. This bioinformatic pipeline was designed to define high‐confidence BICC1 interacting partners, applying a combined filtering strategy as described in previous interactome studies [26, 27]. Initially, to define a highly probable protein partner of BICC1, proteins listed were required to have at least two unique peptides detected in a minimum of two technical replicates and to be absent from Flag‐tagged GFP controls. In parallel, statistical scoring of protein–protein interactions was performed using Significance Analysis of INTeractome (SAINT) analysis through the CRAPome platform (version 2.0) [28], which uses a probabilistic framework to assign confidence scores and estimate the likelihood of true interactions relative to background contaminants. For SAINT analysis, GFP (−) controls were used for BICC1 (−) conditions (FL and ΔSAM), while GFP (+) controls were used for BICC1 (+) conditions (FL and ΔSAM). Proteins meeting these criteria (1—At least two unique peptides detected in a minimum of two technical and 2—SAINT score values greater than 0.7) were considered the most probable BICC1 partners.

Also, BioGRID database was accessed for the identification of already reported protein interactors of BICC1 [22]. Protein–protein interaction networks (PPIN) were generated using the STRING database [29] and visualized in Cytoscape (v3.10.1) [30]. STRING networks were constructed using a confidence score cutoff of 0.4 (medium confidence), considering only physical interactions. Node sizes were displayed according to the degree of connections, and the disconnected nodes were not excluded from the network. The functional enrichment in the PPINs was performed using the functional annotation determined by STRING. Only terms with FDR < 0.05 were considered. Gene Ontology (GO) enrichment analysis was performed using the g:Profiler tool [31]. For each experimental condition, lists of identified proteins were independently queried against the GO Biological Process (GO:BP) and Cellular Component (GO:CC) databases. Enriched terms were filtered based on statistical significance (adjusted *p*‐value < 0.05). The top 10 enriched terms per condition were selected according to the lowest *p*‐values.

### 2.10 Comparison of Immunoblotting and LC–MS/MS Interaction Profiles

Immunoblotting band intensities were quantified using ImageJ software (Fiji version). Original blot images were converted to 8‐bit grayscale, and integrated band densities were measured after background subtraction using the Gel Analysis tool. To account for differences in immunoprecipitation efficiency, target protein signals were normalized to the corresponding immunoprecipitated BICC1 band intensity (FL (−) condition), which was used as the reference condition for comparison across samples since this condition represents the full‐length BICC1 construct under basal conditions, without RNase treatment, and therefore serves as the most biologically representative and experimentally stable interaction profile for relative normalization across conditions. LC–MS/MS values were obtained from normalized LFQ intensity measurements generated by MaxQuant and were normalized relative to BICC1 LFQ intensity values (FL (−) condition) before comparison between orthogonal approaches. The normalized immunoblotting and LC–MS/MS values were plotted using logarithmic scales to facilitate visualization of the dynamic range of interaction signals. The missing values in LC–MS/MS data (values equal zero) were replaced with a small pseudovalue of 1 × 10^−6^ exclusively for log‐scale visualization. In Table S11, we included the normalized immunoblotting densitometry values and corresponding normalized LC–MS/MS LFQ intensity measurements.

### 2.11 Induction of Cellular Stress With Arsenite

HEK293T cells were cultured on poly‐L‐lysine–coated coverslips (São Paulo, Brazil, Sigma, #P6282) in 24‐well plates and transiently transfected with FL or the ΔSAM constructs; non‐transfected cells were used as controls. All conditions were performed in technical triplicate. After 48 h, cells were fixed in 4% paraformaldehyde (PFA) in 0.1 M phosphate buffer (pH 7.4) for 20 min at RT and stored in 1× Phosphate‐Buffered Saline (PBS) at 4°C in the dark. To evaluate subcellular localization under stress, cells overexpressing FL or ΔSAM were treated with 0.5 mM sodium arsenite (NaAsO_2_) for 1 h before fixation with 4% PFA. Fixed cells were permeabilized with 0.1% Triton X‐100 in PBS (PBS‐t) for 15 min and blocked with 2.5% BSA in PBS for 4 h. Transfected cells were incubated with primary antibodies at RT: anti‐Flag M2 and anti‐G3BP1 (see Table S2). After three washes with PBS for 5 min at RT, cells were incubated in the dark with secondary antibodies: FITC‐conjugated anti‐rabbit IgG (São Paulo, Brazil, Proteintech, #SA00003‐2, 1:100) and TRITC‐conjugated goat anti‐mouse IgG (Proteintech, #SA00007‐1, 1:100) for 30 min. Coverslips were mounted with ProLong Gold Antifade with DAPI (Thermo Scientific, #P36931), and images acquired on a Leica TCS SP5 II confocal microscope (LaCTAD, UNICAMP) using 40X and 100X (oil immersion) objectives.

### 2.12 Immunofluorescence for BICC1 Co‐Localization With Protein Partners

Cells were plated (8 × 10^4^ cells/mL) in 24‐well plates and incubated for 24 h with 5% CO_2_ at 37°C before the beginning of treatments. Each well contained a previously autoclaved circular slide, pretreated with Poly‐D‐Lysine (Thermo Scientific, #A3890401). All conditions were performed in technical triplicate. At 48 h after transfections, cells were fixed in 4% PFA in PBS for 20 min at RT. Next, cells were washed three times with PBS and permeabilized with PBS‐t for 1 h at RT. Afterward, cells were incubated with primary antibodies diluted in PBS‐t, according to the conditions described in Table S2. After the primary incubation, cells were washed with PBS five times for 5 min at RT. The secondary antibody was diluted in PBS with DAPI solution (1 mg/mL) added at a final concentration of 1:1000 (1 μg/mg), and cells were incubated with it for 1 h at RT and protected from light. After that, cells were washed with PBS five times for 5 min at RT. The treated cell slides were assembled using ProLong Gold Antifade Mountant with DAPI (Thermo Scientific, #P36941). Immunostained slides were imaged using an Elyra PS.1 Inverted Microscopy (Superresolution Structured Illumination Microscopy) (INFABIC, UNICAMP) fitted with a 63×/1.4 NA glycerol objective.

Due to compatibility constraints between the host species of the primary antibodies, different strategies were used for BICC1 detection and its protein partners. When compatible, an anti‐BICC1 antibody was preferentially used to detect both endogenous and ectopically expressed BICC1. In cases of incompatibility, BICC1 was detected using an anti‐Flag antibody targeting ectopically expressed Flag‐tagged constructs.

### 2.13 Image Processing and Calculation of the Co‐Localization Coefficient

Images were imported into the free image processing software called ImageJ. Brightness/contrast (linear variations) were adjusted, and the background was subtracted using the plugin “subtract background” and the option “disable smoothing.” The same setting of minimum and maximum intensities was used in experimental images and their respective control of conjugated only. Co‐localization analysis was performed using the Coloc 2 plugin in Fiji (ImageJ). Regions of interest (ROIs) corresponding to individual cells were manually defined and consistently applied across both channels using the ROI Manager. Pearson's correlation coefficient (PCC) was calculated to assess the degree of correlation between fluorescence signals, ranging from −1 (complete negative correlation) to 1 (complete positive correlation). Automatic thresholding was applied using the Costes method to minimize background contribution. A minimum of *n* = 10 cells per condition was analyzed, and the mean PCC and standard deviation (SD) were calculated across cells (Table S12).

### 2.14 Kaplan–Meier Survival Analysis

Survival analysis was performed using the Kaplan–Meier Plotter online tool (Pan‐cancer RNA‐seq dataset) [32], which integrates gene expression and clinical data from publicly available cancer cohorts. Patients with pancreatic ductal adenocarcinoma (*n* = 177 datasets) were selected, and overall survival (OS) was used as the clinical endpoint. Patients were stratified into high and low expression groups based on the median expression of each gene (BICC1, PRMT5, IGF2BP1, and STK38). OS was evaluated using Kaplan–Meier curves, and differences between groups were assessed using the log‐rank test. Hazard ratios (HRs) and corresponding 95% confidence intervals were calculated to estimate the relative risk of death between the high and low expression groups. A *p*‐value < 0.05 was considered statistically significant. Clinical variables such as race and gender were considered in this analysis. However, due to limited sample size in non‐White groups (Asian and Black/African American), statistical analyses were restricted to the White cohort (*n* = 5485 patients), which was further stratified by sex (female and male).

## 3 Results

### 3.1 Confirmation of BICC1 Co‐IP Upon RNA and SAM Domain Dependence and Identification of Proteins Detected by Mass Spectrometry

**FIGURE 1:**
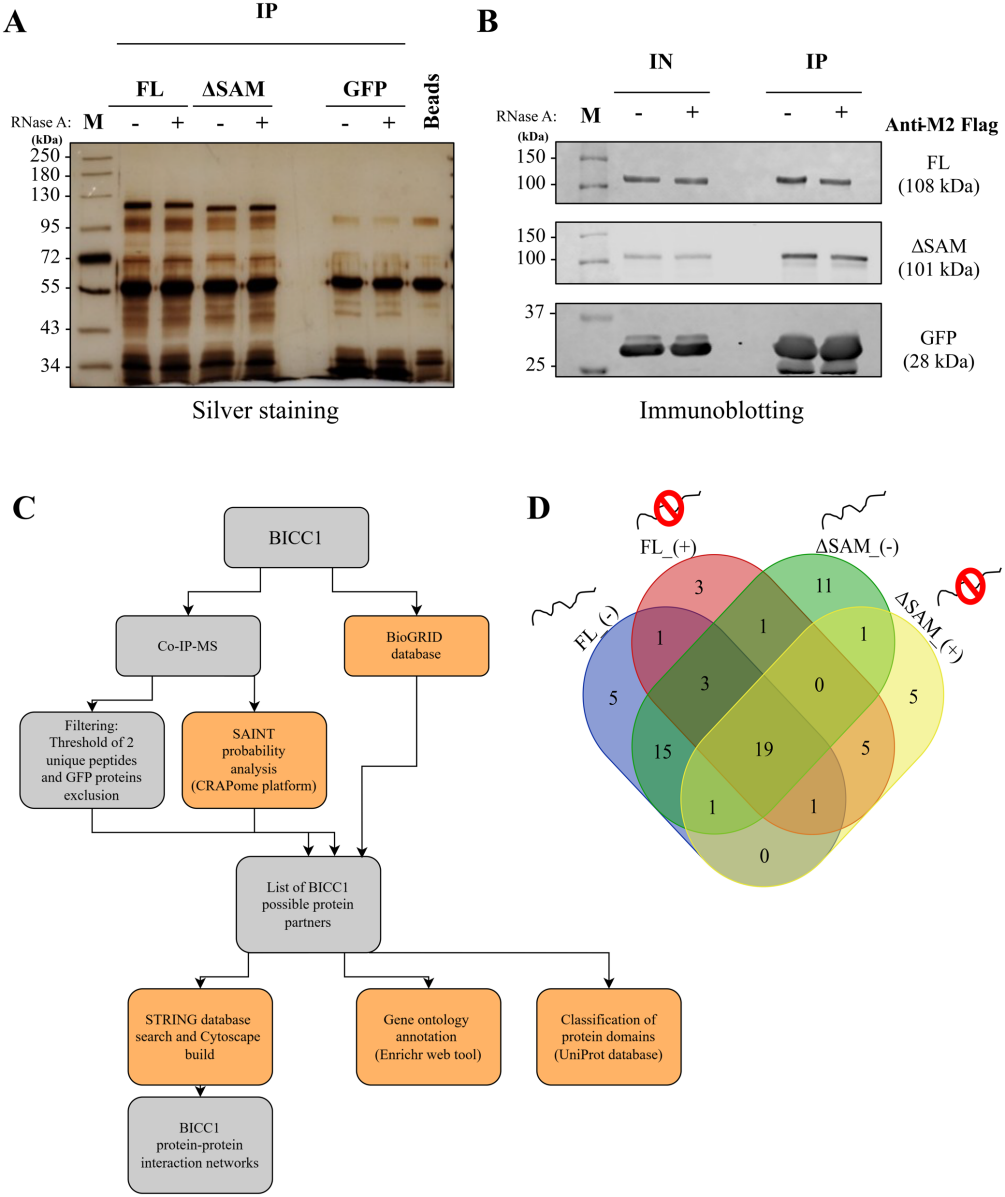
Co‐immunoprecipitation of BICC1‐Flag (FL and ΔSAM) for interactome analyses upon RNA and SAM domain dependence. (A) Silver staining of proteins co‐immunoprecipitated with BICC1 protein, followed by Co‐IPs of the experimental controls: GFP protein expression and only the incubation of the magnetic beads. (B) Immunoblotting of Co‐IPs with anti‐flag antibody, confirming the Co‐IP of BICC1 constructs and GFP. (C) Bioinformatic pipeline followed for Co‐IP‐MS results analysis. (D) Venn diagram of the BICC1 interacting proteins using a threshold of 2 unique peptides present in at least two replicates and exclusion of proteins present in GFP conditions. IN = Input (the sample before immunoprecipitation, qs 20 μg BSA‐Eq); IP = Immunoprecipitated (20% of the eluate = 2 μL); (−) means addition of recombinant RNAase inhibitor; (+) means addition of 15 μg of recombinant RNase A. The same input was used for (−) and (+) treatments.

After confirming the cloning of BICC1 (FL and ΔSAM) constructs and their overexpression in HEK293T cells (Figure S2), Co‐IP assays were performed to determine the interactome. Four experimental conditions were analyzed: (1) Full length (FL) upon RNA presence (−); (2) Full length upon RNA depletion, performed with RNase A treatment (+) (Table S3); (3) Deletion of the SAM domain (ΔSAM) upon RNA presence (−) (Table S4), and (4) ΔSAM upon RNA depletion, performed with RNase A treatment (+) (Table S5). RNase A concentrations were optimized based on RNA digestion assays (Figure S3). After Co‐IP, we observed that most detected proteins were specifically associated with BICC1‐containing complexes. As indicated, Co‐IP efficiency and specificity were assessed by silver nitrate staining (Figure 1A) and immunoblotting (Figure 1B). Eluates from BICC1 Co‐IPs contained a greater number of protein bands compared with GFP control Co‐IPs and M2‐Flag beads alone (Tables S6 and S7). These findings were confirmed by mass spectrometry analysis.

Proteomic data were processed using a standardized bioinformatic pipeline (Figure 1C), resulting in the detection of distinct sets of interacting proteins across conditions. Overlaps and condition‐specific interactions were visualized using a Venn diagram (Figure 1D). In total, 71 proteins were identified as potential BICC1‐associated partners. RNase A treatment consistently reduced the number of co‐immunoprecipitated proteins, indicating that RNA contributes to the composition of BICC1‐associated complexes. In contrast, deletion of the SAM domain was associated with an increased number of interacting proteins under RNA‐preserved conditions, suggesting altered interaction dynamics in the absence of this domain.

To further assess RNA dependency, the identified proteins were classified based on the presence of known RNA‐binding domains (RBDs). Conditions preserving RNA integrity revealed a total of 23 proteins containing RBDs, of which 10 were identified in the FL (−) condition and 13 in the ΔSAM (−) condition. In comparison to the (+) conditions, these datasets showed a higher proportion (100%) of proteins harboring RBDs, including KH, RRM, DDX, and CSD domains, with five proteins detected exclusively under RNA‐preserving conditions: FL (−) ‐ PCBP1 and ΔSAM (−) ‐ IGF2BP1, KHDRBS1, PABPC1 and HNRNPD (Table S9). Together, these data support a role for RNA in shaping the BICC1 interactome.

### 3.2 BICC1 Co‐Immunoprecipitates With Proteins Associated With Biomolecular Condensates and Co‐Localizes With Stress Granules

**FIGURE 2:**
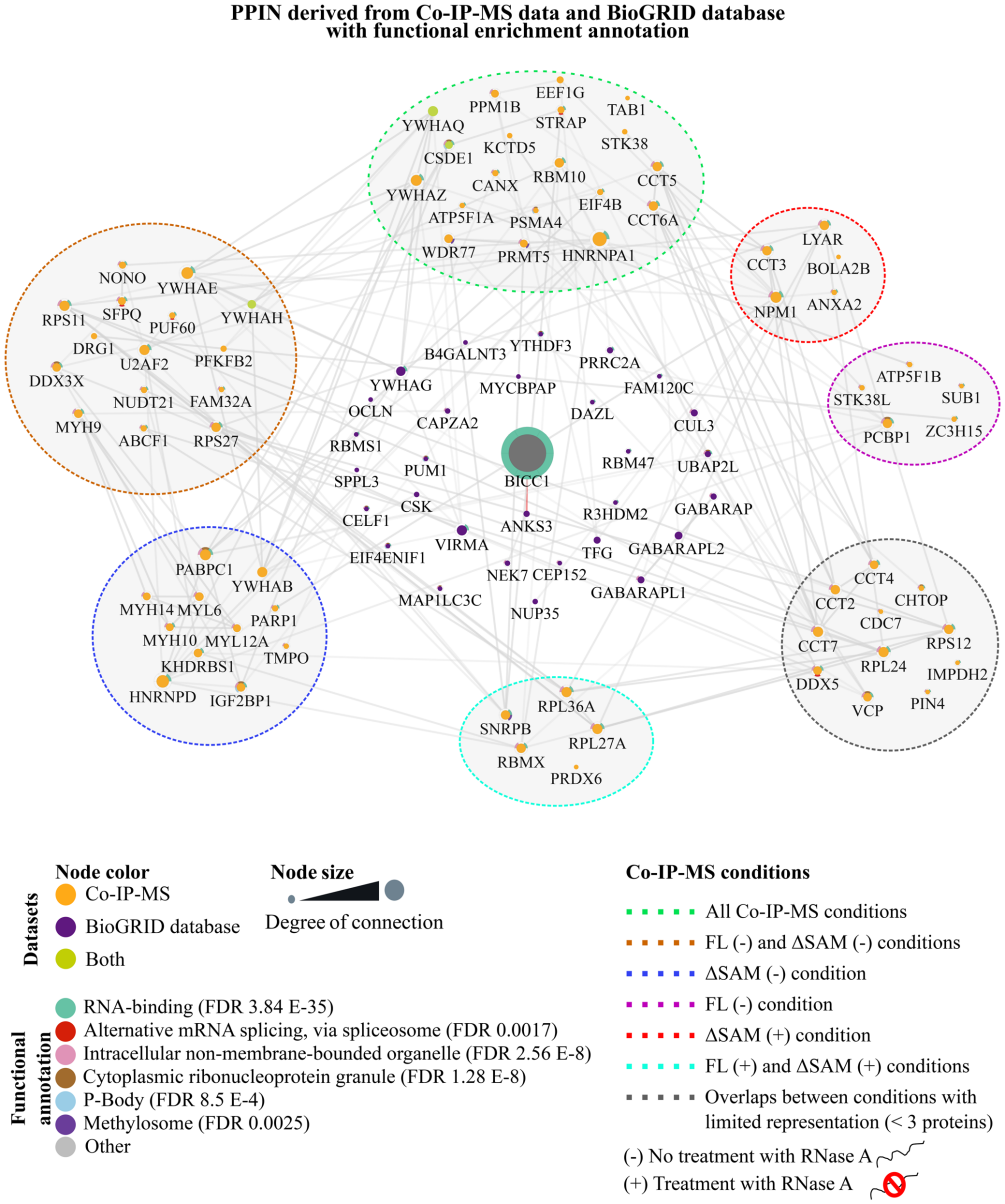
PPIN of all proteins identified in the four different BICC1 Co‐IPs experimental conditions (*n* = 71) and proteins present in the BioGRID database (*n* = 35). PPIN nodes were colored according to proteins identified in this Co‐IP‐MS study, proteins exclusive to the BioGRID database, and proteins present in both conditions. Also, the nodes were colored according to the enriched functional enrichment annotation determined by STRING (FDR < 0.05) and grouped according to the Co‐IP‐MS conditions. The networks were constructed using Cytoscape and the STRING plugin. The node sizes were displayed according to the degree of connections. PPINs were classified according to the physical subnetwork and edges with medium confidence: 0,400. Enrichment *p*‐value = 1.0E‐16.

A general PPIN was generated using all proteins identified under BICC1 Co‐IP conditions (*n* = 71), together with proteins reported in the BioGRID database (*n* = 35, last updated September 2025). In addition, the network was stratified according to the different Co‐IP‐MS experimental conditions to enable condition‐specific visualization of interactions (Figure 2). An overlap between both datasets was observed for CSDE1 (SAINT score > 0.99), YWHAQ (SAINT score > 0.2), and YWHAH (SAINT score > 0.5) (highlighted in Figure 2). Notably, these proteins have also been previously identified in high‐throughput interaction studies, further supporting their association with BICC1 [33, 34]. The high SAINT score observed for CSDE1 indicates a highly confident interaction, whereas the lower scores for YWHAH and YWHAQ may reflect more transient or context‐dependent interactions within the BICC1 interactome. Moreover, several enriched functional annotations with a false discovery rate (FDR) < 0.05 were mapped onto the PPIN (Figure 2). These included biological processes related to RNA binding activity, such as mRNA splicing, and cellular components linked to the PRMT5 methylosome complex and to membraneless organelles. The latter are typically [33, 34] formed through liquid–liquid phase separation (LLPS), giving rise to biomolecular condensates [35, 36].

**FIGURE 3:**
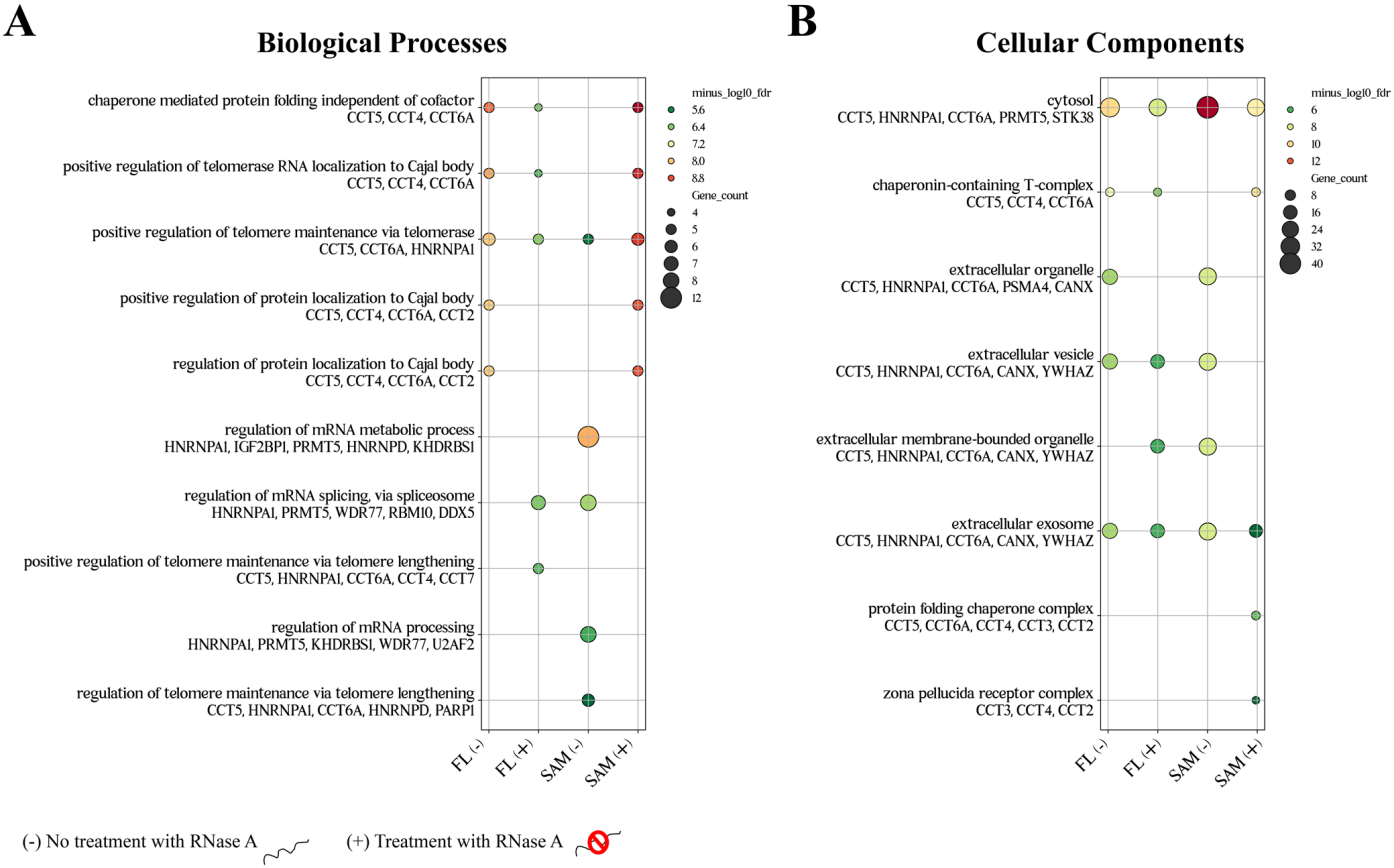
Gene Ontology (GO) enrichment analysis of proteins co‐immunoprecipitated with BICC1 across experimental conditions. Dot plot showing the top 5 enriched Biological Processes (A) and Cellular Components (B) (adjusted *p*‐value < 0.05) identified using the g:Profiler tool [31]. Each dot represents a GO term enriched in a given condition. Dot size indicates the number of proteins associated with each term (gene count), while color represents the enrichment significance (−log10 adjusted *p*‐value). GO terms are ordered based on their overall significance across conditions, and labels include representative shared proteins contributing to each term.

As complementary analyses, GO enrichment was performed on proteins co‐immunoprecipitated under each Co‐IP‐MS condition (Figure 3). Notably, the absence of the SAM domain combined with RNA degradation (ΔSAM (+)) resulted in enrichment of biological processes associated with Cajal bodies, alongside cellular components related to vesicles. In contrast, the ΔSAM condition with RNA preserved (ΔSAM (−)) was predominantly enriched for terms linked to RNA‐related processes, highlighting a shift toward RNA‐associated functions when RNA integrity is maintained. For the full‐length BICC1, both conditions, with and without RNA preservation (FL (−) and FL (+)), consistently showed enrichment for Cajal body–associated processes. Additionally, these conditions were enriched for terms related to protein localization within the nucleus, as well as extracellular exosomes and vesicles (Figure 3).

**FIGURE 4:**
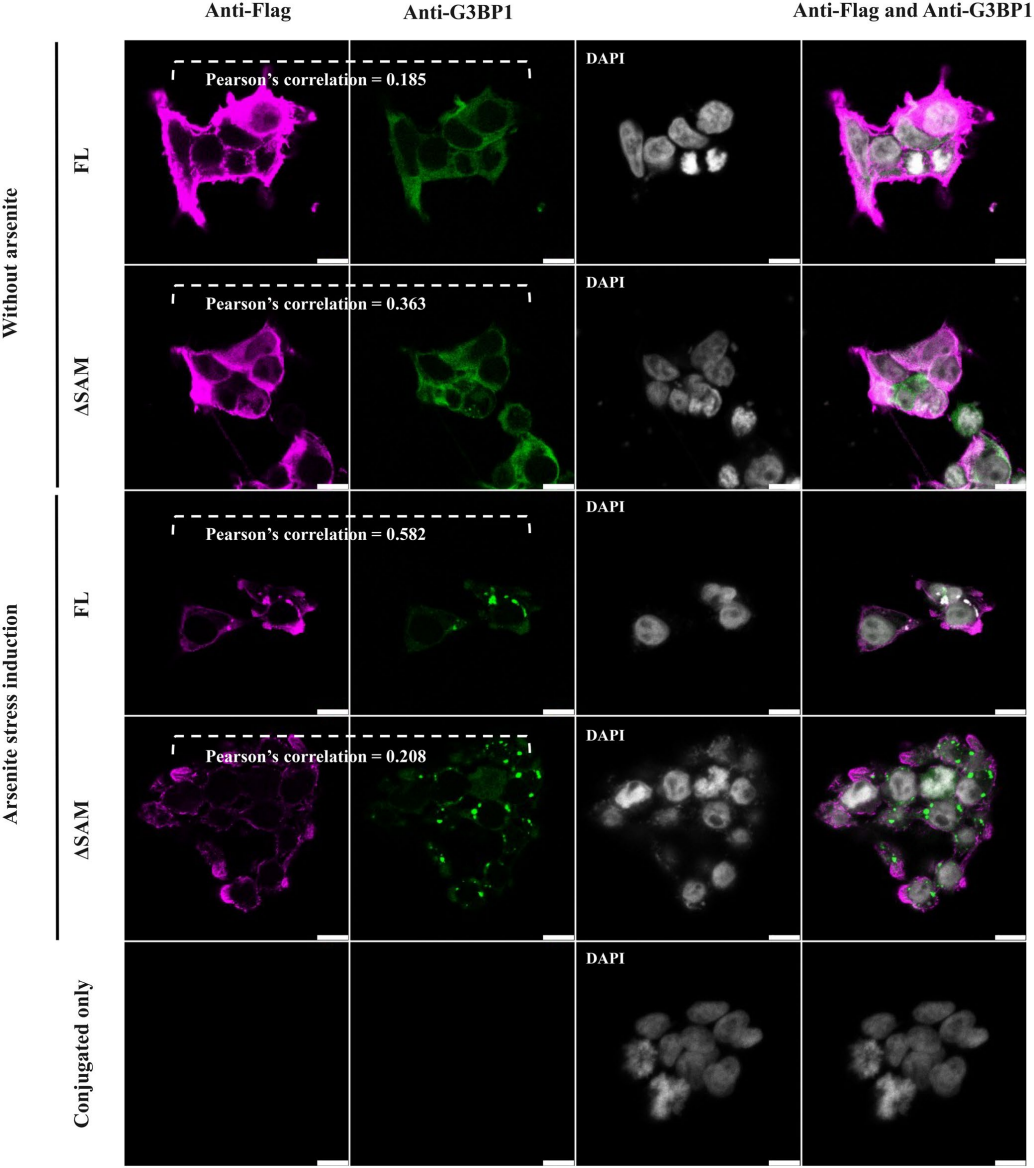
BICC1 is recruited to stress granules over sodium arsenite stress induction in HEK293 cells in a SAM domain‐dependent process. Confocal microscopy images with a 100× objective (in immersion oil) and 2× zoom in. Cellular stress induction with sodium arsenite (NaAsO2 0.5 mM for 1 h before fixation) causes stress granule maturation with BICC1 recruitment and colocalization with G3BP1 (stress‐granule marker). The SAM domain deletion (ΔSAM) compromises this process. Experiments were performed in triplicate for each condition, and a total of 10 cell regions were analyzed to calculate the average Pearson's correlation coefficient, which is highlighted in each experimental condition. Scale bar = 10 μm.

The protein‐containing complexes (Cajal bodies, SG, and P‐bodies) are formed during LLPS, which is an activity in the cell important for survival during stress conditions [36]. Indeed, the BICC1 protein sequence, when submitted to a sequence‐based web tool for query and prediction of potential phase separation proteins (PSPredictor) [37], showed a high positive score of 0.9919. PSPredictor uses machine learning to predict potential phase‐separating proteins based on curated data from the Liquid–Liquid Phase Separation Database (LLPSDB) [37]. Scores closer to 1 indicate a higher likelihood that the protein forms liquid condensate structures [37]. In addition, the proteins identified in our Co‐IP experiments were cross‐referenced with the Database of proteins related to Liquid–Liquid Phase Separation (DrLLPS) [38]. Of the 71 proteins detected, 51 were represented in the database, including 10 proteins with reported evidence in P‐bodies, 27 in SGs, and the remainder associated with other categories of evidence, as nucleolus, postsynaptic density, and Cajal bodies (Table S10). Therefore, our results showed that the BICC1 interactome was strongly associated with biomolecular condensate proteins, mostly SGs. To confirm this, cellular assays were performed by overexpressing BICC1 constructs (FL and ΔSAM) and inducing stress granule formation with sodium arsenite. Immunofluorescence staining of G3BP1, a marker of SGs, revealed BICC1 co‐localization. Interestingly, this co‐localization appeared to depend on the SAM domain (Figures 4 and S4). Indeed, the co‐localization of BICC1 in SGs was already reported in previous studies [10, 39].

### 3.3 Splicing Proteins in BICC1 Co‐IPs Are Dependent on the Presence of RNA

**FIGURE 5:**
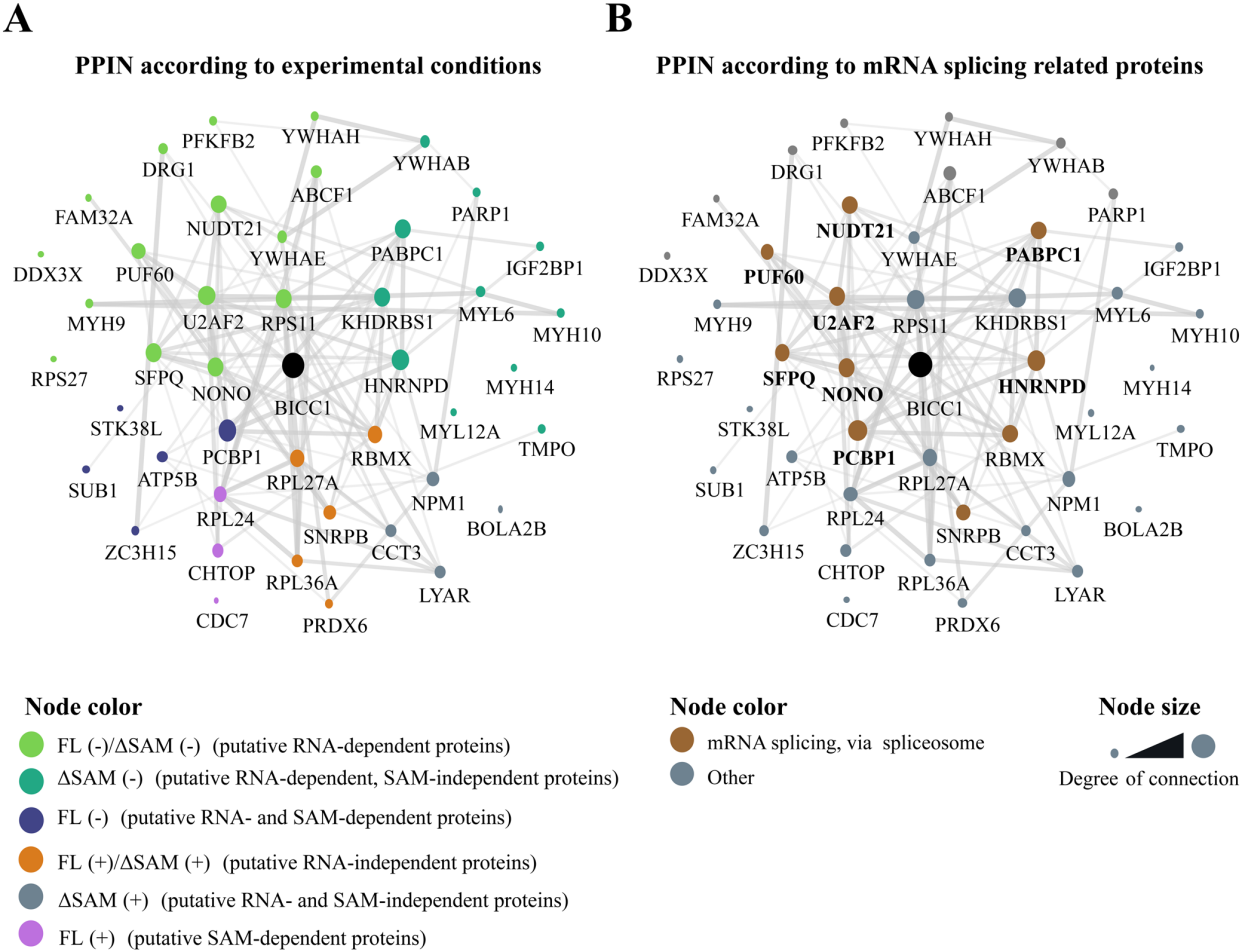
Proteins related to splicing were enriched in the Co‐IPs upon RNA dependence. (A) PPIN of proteins exclusively identified in each separated BICC1 (FL and ΔSAM) Co‐IPs, in the presence or absence of RNase A treatment. (B) Splicing‐related proteins highlighted in the PPIN. PPINs were classified according to the physical subnetwork and edges with medium confidence: 0,400. Enrichment *p*‐value = 1.0E‐16. The node sizes were displayed according to the degree of connections. Proteins highlighted in bold were exclusive to RNA‐dependent conditions. The networks were constructed using Cytoscape v3.10.1.

The connections between the proteins that were exclusive in each of the four Co‐IP conditions, as shown in the Venn diagram of Figure 1D, were investigated, aiming to identify potential biological processes enriched in the determined conditions. In Figure 5A, there were six different conditions analyzed: (1) FL (−)/ΔSAM (−) (probable RNA dependent proteins); (2) ΔSAM (−) (probable RNA dependent and SAM domain independent proteins); (3) FL (−) (probable RNA and SAM domain‐dependent proteins); (4) FL (+)/ΔSAM (+) (probable RNA independent proteins); (5) ΔSAM (+) (probable RNA and SAM domain independent proteins), and (6) FL (+) (probable SAM domain‐dependent proteins).

The proteins with the highest degree of connections (> 10 connections) were PCBP1, U2AF2, and HNRNPD, and were also exclusively present in the Co‐IP conditions without RNase A treatment. In this PPIN, it was also possible to observe that most of the proteins and connections are from Co‐IP conditions without RNase A treatment, suggesting the importance of RNA presence for the BICC1 interactome. In addition, in the same PPIN, splicing‐related proteins were highlighted with a different node color (Figure 5B). Interestingly, eight of ten were in the conditions of RNA dependence: PUF60, NUDT21, U2AF2, SFPQ, NONO, PCBP1, PABPC1, and HNRNPD (highlighted in bold in Figure 5B). Together, these findings demonstrate that Co‐IP combined with mass spectrometry provides a consistent view of the BICC1 interactome and that RNase A treatment markedly reduces RNA‐mediated protein–protein interactions, emphasizing the importance of RNA in stabilizing BICC1‐associated protein complexes.

### 3.4 Orthogonal Validation of BICC1 Protein Partners

According to PPIN and GO analysis, some proteins were considered for further investigation of BICC1 interaction: PRMT5 (SAINT score = 1), STK38 (SAINT score > 0.98), IGF2BP1 (SAINT score = 0.7), and PARP1 (SAINT score = 0.79). These selected proteins showed high‐confidence interactions with BICC1, supported by SAINT probability scores greater than 0.7. Functionally, PRMT5 is involved in arginine methylation and regulation of RNA splicing and gene expression [40]; STK38 (NDR1 kinase) participates in apoptosis and autophagy signaling [41]; PARP1 plays a key role in DNA damage response and chromatin remodeling [42]; and IGF2BP1 regulates mRNA stability and translation [43]. Notably, PRMT5 has also been previously implicated in glioblastoma in studies from our group [44], further supporting its relevance in this context. Together, these results highlight PRMT5, STK38, PARP1, and IGF2BP1 as prominent and high‐confidence BICC1‐associated proteins linked to the major functional pathways identified in our analysis.

**FIGURE 6:**
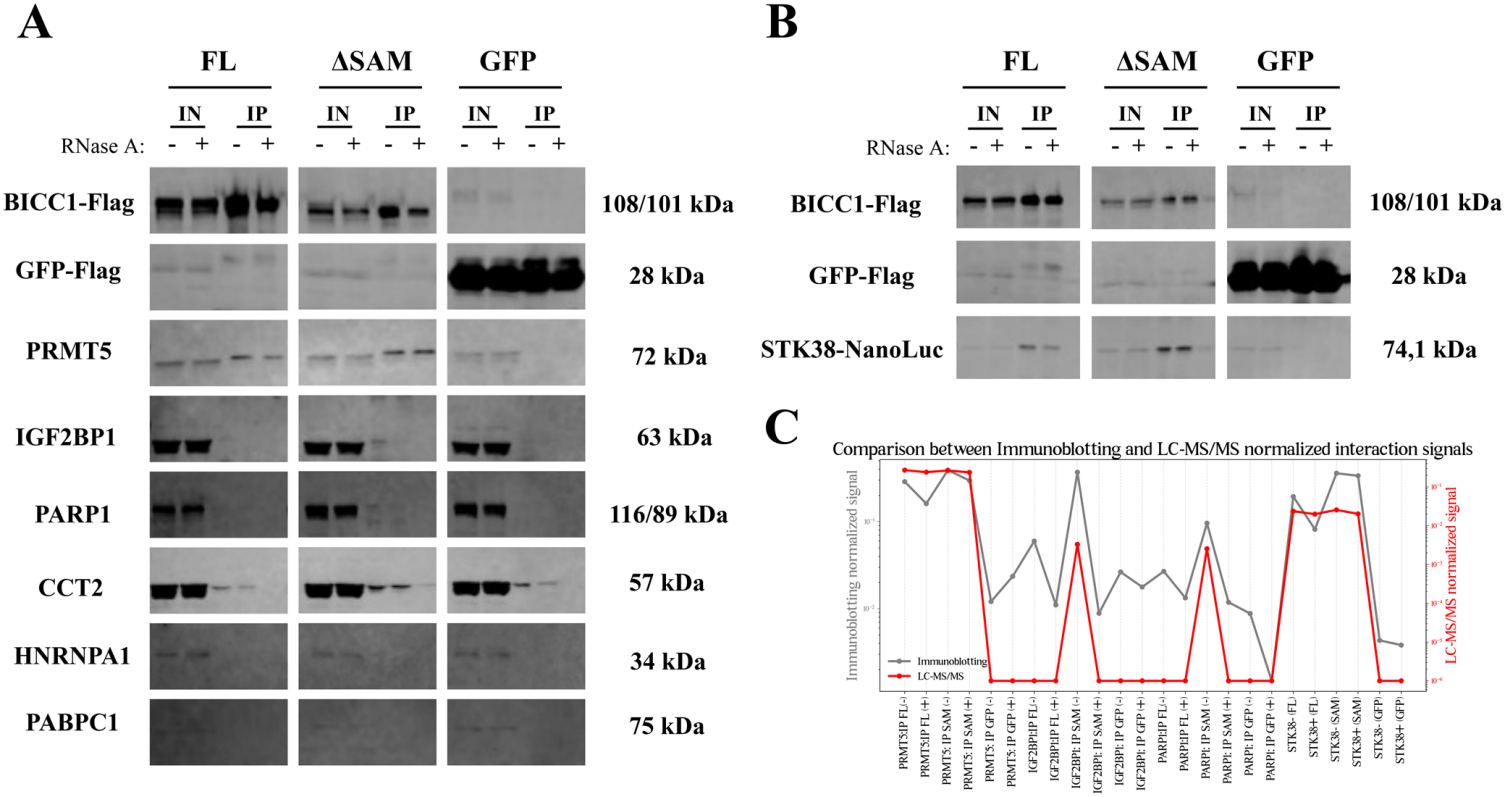
Co‐IP of BICC1 followed by Immunoblotting reveals interaction with PRMT5, IGF2BP1, PARP1, and STK38. (A) Overexpression of BICC1 constructs (FL and ΔSAM) followed by Co‐IP with anti‐Flag magnetic beads; (B) Overexpression of BICC1 constructs (FL and ΔSAM) and STK38 construct followed by Co‐IP with anti‐Flag magnetic beads. IN = input (20 μg BSA‐Eq of total protein); IP = immunoprecipitation (Final volume of IP elution = 20 μL); (−) means addition of recombinant RNAase inhibitor; (+) means addition of 15 μg of recombinant RNase A. The same input was used for (−) and (+) treatments. (C) Normalized interaction signals obtained by immunoblotting (independent replicate) and LC–MS/MS analyses (*n* = 3 technical replicates) for PRMT5, IGF2BP1, PARP1, and STK38 under FL, SAM, and GFP conditions. Immunoblotting values were calculated by normalizing target protein immunoprecipitation band intensities to the FL (−) immunoprecipitation signal. LC–MS/MS values correspond to normalized LFQ intensity ratios relative to FL (−) LFQ intensity. Dual logarithmic y‐axes were used to facilitate visualization of the broad dynamic range and low‐abundance interaction signals. Near‐zero LC–MS/MS values were replaced with 1 × 10^−6^ for log‐scale visualization purposes only. The analyses demonstrate qualitative agreement between orthogonal analytical approaches across the evaluated interaction candidates.

Immunoblotting confirmed that PRMT5 co‐immunoprecipitates with BICC1, but not with GFP (Figure 6A). Notably, PRMT5 was enriched in the BICC1 Co‐IP compared to the input, indicating a specific interaction. This association seems to be independent of SAM and RNA, suggesting that other domains are likely to mediate this interaction. A slight shift in the apparent molecular weight of PRMT5 was observed between input and co‐immunoprecipitated samples. This is consistent with its known association with multiprotein complexes [45, 46, 47, 48], such as WDR77 protein, which can affect electrophoretic mobility. Importantly, the detected band remained within the expected molecular weight range (~72 kDa), supporting its specificity. In addition to this methyltransferase, BICC1 Co‐IP also confirmed an interaction with IGF2BP1 (Figure 6A), which was stronger in the presence of RNA. For instance, IGF2BP1 was primarily detected in the ΔSAM (−) Co‐IP condition, consistent with mass spectrometry data showing this protein only under this condition (Table S5). As already described in Table S3, IGF2BP1 is an RBP that contains KH domains; thus, its predominant presence in conditions with RNA dependence and with the KH domains of BICC1 (ΔSAM (−)) shows the specificity of this protein to RNA‐binding functions in the cell. Also, PARP1 was predominantly observed in the ΔSAM (−) Co‐IP, with only a weak signal in the other Co‐IP conditions, consistent with the Co‐IP MS data, which detected PARP1 exclusively in this condition (Table S5).

In parallel, STK38 interaction was assessed using a NanoLuciferase‐tagged construct (19.1 kDa), selected due to its small size and reduced steric hindrance. This approach was specifically applied to STK38 to enhance detection sensitivity. Immunoblotting (Figure 6B) confirmed that STK38 co‐immunoprecipitated with BICC1, with IP bands more enriched than the input. In contrast, other proteins were explored: PABPC1 (SAINT score = 0.82), HNRNPA1 (SAINT score > 0.72), and CCT2 (SAINT score > 0.24); however, they did not show specific co‐immunoprecipitation with BICC1 under the conditions tested. This may reflect transient or RNA‐dependent interactions, as well as technical limitations such as antibody sensitivity. CCT2 was also detected in GFP‐Flag control eluates, indicating nonspecific binding under these conditions. Consistently, this protein exhibited low SAINT probability scores, further supporting its classification as a low‐confidence interactor. As an orthogonal validation of the immunoblotting approach, we quantitatively compared the densitometric intensities of immunoblot bands corresponding to validated BICC1‐interacting proteins with the normalized peptide intensity profiles obtained by LC–MS/MS analysis (Figure 6C). The results revealed a consistent interaction pattern across both analytical and independent approaches.

Next, we investigated the cellular co‐localization of BICC1 with selected partners identified by mass spectrometry and confirmed by immunoblotting. HEK293T cells were transfected with the full‐length BICC1 construct and analyzed by immunofluorescence. Although a high degree of overlap between endogenous BICC1 and Flag‐BICC1 was observed, the Pearson correlation coefficient (average 0.749, Table S12) did not reach 1. This is likely due to heterogeneous transfection efficiency, resulting in a mixed population of transfected and non‐transfected cells, as well as differences in the distribution of endogenous versus overexpressed protein. After validating the efficiency of BICC1 and Flag antibodies for proper detection (Figure S5), the RBP was observed to co‐localize with PRMT5 at specific cellular sites, suggesting a transient interaction between these proteins (Figure S6). Co‐localization with PARP1 was detected in a few cells during the prophase/metaphase stages of the cell cycle (white dots in Figure S7), with a positive Pearson's correlation coefficient of 0.87. In contrast, other cell cycle stages showed a negative correlation. Table S12 summarizes Pearson's correlation coefficients for the analyzed cells (*n* = 10 cells per condition), along with the average values and standard deviations.

### 3.5 Prognostic Significance of BICC1 and Its Interacting Partners in Pancreatic Cancer

To extend our findings to a clinical exploratory context, we investigated whether the expression of BICC1 and its validated interaction partners was associated with patient survival using Kaplan–Meier analysis [49]. Given the reported involvement of BICC1 dysregulation in pancreatic cancer [14, 20, 50], we focused on pancreatic ductal adenocarcinoma datasets. Survival analysis was performed using publicly available pancreatic ductal adenocarcinoma datasets (TCGA). Patients were stratified based on gene expression levels (high vs. low expression), and overall survival was analyzed using Kaplan–Meier estimates.

**FIGURE 7:**
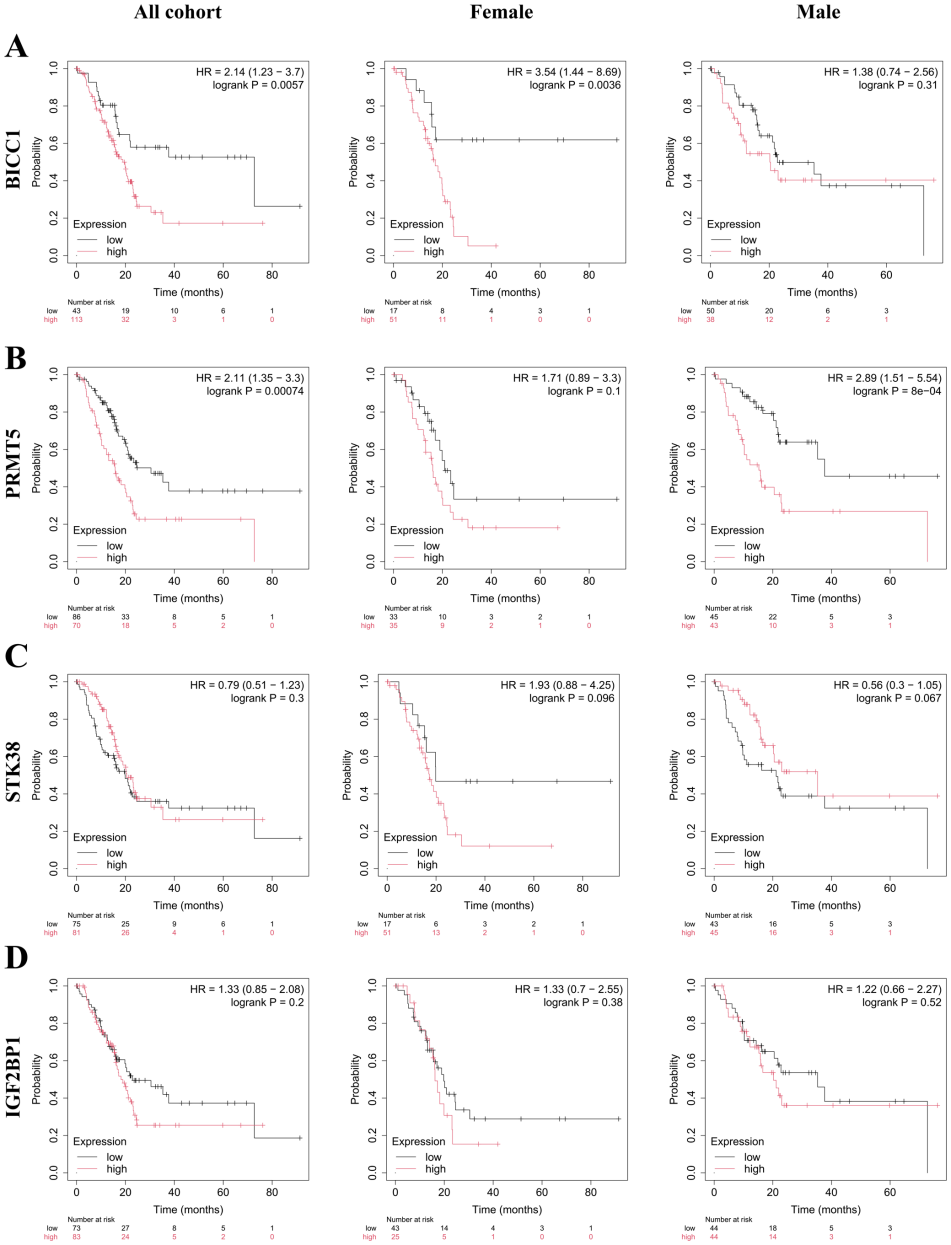
Kaplan–Meier analysis. Comparing survival of pancreatic ductal adenocarcinoma according to BICC1 (A), PRMT5 (B), STK38 (C), and IGF2BP1 (D) expression. The x‐axis represents time (in months) and the y‐axis represents the probability of survival. Sudden drops indicate death, and the presence of short vertical dashes indicates censored patients (those who dropped out of the study or were still alive at the last follow‐up). Red curve (High) represents patients with high expression of the gene; Black curve (Low) represents patients with low expression of the same gene group. HR (Hazard Ratio) compares the risk of death between two groups (here, High vs. Low expression). HR = 1 means that there is no difference between the groups; HR > 1 means that the “High” group (high expression) has a higher risk of death than the “Low” group; HR < 1 means that the “High” group has a lower risk of death than the “Low” group. Logrank *p* < 0.05 means that there is a significant difference between groups (high and low).

High expression of BICC1 and PRMT5 was significantly associated with reduced patient survival (log‐rank *p* < 0.05), with hazard ratios greater than 2.0, indicating a strong association with increased risk of death (Figure 7A,B). Notably, stratification by sex revealed distinct patterns: the prognostic impact of BICC1 was more pronounced in female patients, whereas PRMT5 showed a stronger and significant association with poor survival, in male patients, suggesting sex‐specific effects. In contrast, IGF2BP1 and STK38 exhibited trends toward altered survival outcomes; however, these associations did not reach statistical significance (log‐rank *p* > 0.05) (Figure 7A,C,D). Interestingly, STK38 displayed opposite tendencies between sexes, indicating a potential context‐dependent role.

Overall, these results suggest that BICC1 and PRMT5 are robust prognostic markers associated with poor survival in pancreatic cancer, with evidence of sex‐dependent effects, while IGF2BP1 and STK38 may represent potential candidates that warrant further investigation.

## 4 Discussion

In this study, we characterized the BICC1 interactome using Co‐IP‐MS, identifying a set of novel protein partners. PPIN and GO annotation revealed enrichment in pathways related to biomolecular condensates, mRNA splicing, and methylosome complex. Importantly, our findings indicate that high expression levels of BICC1 and validated protein partners by immunoblotting (PRMT5, IGF2BP1, and STK38) are associated with poorer survival outcomes in pancreatic cancer patients, suggesting a potential oncogenic role of BICC1 in this context. Overall, these results provide new insights into the intracellular functions of BICC1 and its protein partners, highlighting their possible relevance in pancreatic tumor biology.

Here, we identified 71 proteins that specifically interact with BICC1, and just a few of them were previously reported in high‐throughput studies (CSDE1, YWHAQ, and YWHAH) [33, 34]. The investigation of BICC1 interactome upon RNA or SAM domain conditions revealed different arrangements of protein‐partners, suggesting a complex mechanism of protein interactions. It is well known that KH domains are primarily responsible for RNA binding, whereas SAM domains are usually involved in protein–protein interactions, and less frequently, in RNA binding [51]. However, this study reported the presence of BICC1 partners even in the complete absence of SAM domains and RNA, raising the possibility of new mechanisms of interaction. Similarly, earlier studies have demonstrated that BICC1 interacts with proteins from the CCR4‐Not complex, at least in part, through KH domains, and this interaction is independent of RNA presence or the SAM domain [25]. These new findings reinforce that KH domains could also act as protein‐binding sites. Furthermore, our data indicate that most BICC1‐binding proteins are identified in the presence of RNAs, suggesting that BICC1 and its primary binding proteins share the regulation of specific RNA targets.

Interestingly, an increased number of interacting proteins was observed with the ΔSAM construct, suggesting an altered interaction dynamics in the absence of the SAM domain. This result may reflect increased accessibility of protein‐binding surfaces that are normally masked in the full‐length protein. In addition, since the SAM domain mediates BICC1 polymerization, its deletion may disrupt higher‐order assemblies and expose interaction interfaces [52, 53]. However, we cannot exclude that structural alterations in the truncated protein may contribute to nonphysiological interactions.

Next, using PPIN and GO‐term analysis to link the BICC1 interactome to biological functions and pathways, we observed that most of the enriched BICC1 partners were proteins involved in splicing regulation. According to this analysis, ten proteins were associated with mRNA splicing via the spliceosome, and eight of them were exclusive to RNA‐dependent Co‐IP conditions. In addition, our dataset included several RBPs with established roles in splicing and RNA processing, such as PUF60, NONO, PCBP1, PABPC1, and U2AF2. Notably, PUF60 and U2AF2 are core components of splice site recognition, contributing to 3′ splice site selection and spliceosome assembly [54]. NONO has been implicated in coupling transcription and RNA processing, including splicing regulation [55, 56]. Finally, PCBP1 and PABPC1 are classically linked to mRNA stability and translation, but they have also been reported to influence alternative splicing through RNA binding and modulation of transcript fate [57, 58]. However, to date, no studies have reported an association between BICC1 and splicing activity. RNA splicing is crucial for the expression of most human genes, and abnormal splicing activity is associated with various types of cancer [44, 59]. Given that BICC1 is also implicated in cancer and cancer survival [14, 18, 19, 50, 60, 61], it would be interesting to explore the connection between this RBP and RNA splicing within a cancer context in future studies.

Our data also revealed proteins associated with the PRMT5 methylosome complex (PRMT5, WDR77, and SNRPB) in the BICC1 interactome, independent of RNA and SAM domain. The Co‐IP of BICC1 with the methyltransferase PRMT5 was next validated through immunoblotting and immunofluorescence. The methylosome complex, primarily composed of PRMT5 and WDR77, is involved in splicing regulation by methylating Sm proteins, which are essential for spliceosome assembly [40, 48, 62]. Moreover, this complex plays a critical role in cancer development, including glioblastoma [44] and breast cancer [47]. The BICC1‐PRMT5 interaction could be further explored in disease contexts, especially in glioblastoma and pancreatic cancer, since PRMT5 is strongly associated with the development of these diseases [44, 63] and BICC1 was recently pointed out as a potential RBP signature for glioblastoma prognosis and treatment [44] and to modulate tumor progression in pancreatic cancer [14, 20, 50]. Given the central role of PRMT5 in catalyzing posttranslational modifications (PTMs), particularly arginine methylation [40, 48, 62], the interaction between BICC1 and the methylosome complex raises the possibility that BICC1 or its associated partners may be subject to regulatory PTMs. Such PTMs could contribute to the modulation of splicing and cancer‐related pathways, providing an additional layer of regulation to be explored in future studies.

Notably, proteins with cellular localization in biomolecular condensates (e.g., P‐bodies, SGs, and Cajal bodies) were strongly enriched in the PPINs, and BICC1 was co‐localized in SGs in a SAM domain dependent manner. These proteins are characterized as membraneless organelle proteins (MLOs), which are a type of pro‐survival structure formed when cells are exposed to multiple stresses [64]. MLOs do not contain a lipid membrane but rather are assembled from LLPS of biopolymers and protect cells from genetic damage during stress conditions [64]. In total, 52 proteins (including BICC1) were classified in this class. Indeed, BICC1 is reported to be associated with SG when translation is inhibited, and the SAM domain is pointed out as especially important for BICC1 recruitment to RNA‐processing bodies (P‐bodies) [10, 34, 39]. Additionally, IGF2BP1, a well‐characterized RBP involved in mRNA stabilization [43] and known to be localized in SGs [65, 66], was identified as a BICC1 protein partner upon RNA dependence. This finding is consistent with the enrichment of MLOs in the interactome and supports the role of BICC1 in RNA metabolism within biomolecular condensates.

In addition to the new data presented in this study, it is important to acknowledge several limitations. First, the limited number of independent biological replicates reduces the statistical robustness of the identified interaction patterns and therefore the findings should be interpreted primarily as exploratory and hypothesis‐generating observations. Although orthogonal analytical approaches, including immunoblotting validation and immunofluorescence analyses, supported the consistency of several interaction and localization patterns, these analyses do not replace independent biological replication. Accordingly, the BICC1‐associated proteins identified in this study constitute a discovery‐based resource that will benefit from additional biological validation in future studies.

Finally, we did not detect previously reported BICC1 interactors, including ANKS3, ANKS6, and components of the CCR4‐NOT complex [25, 39]. Although these proteins have been described as BICC1 partners in prior studies, their absence in our dataset may reflect context‐dependent interactions, differences in experimental conditions, or technical limitations inherent to Co‐IP–MS approaches, such as the loss of transient or low‐abundance interactions. Also, the supraphysiological protein levels resulting from BICC1 overexpression may increase the risk of detecting artificial interactions. Furthermore, although RNase A treatment was used to distinguish RNA‐dependent from RNA‐independent interactions, and RNA digestion was validated using purified samples, we cannot fully exclude incomplete RNA degradation in complex cell lysates, where associated proteins may partially protect RNA. Consistent with effective RNA disruption, our Co‐IP‐MS data showed a global decrease in the number of proteins identified upon RNase A treatment, including proteins containing RBDs, supporting the loss of RNA‐mediated interactions. However, residual RNA may still contribute to a subset of interactions observed under RNase A (+) conditions.

Therefore, while our dataset provides a useful framework for characterizing the BICC1 interactome in HEK293T cells, further studies using complementary and biologically independent validation approaches will be required to confirm the specificity, reproducibility, and functional relevance of the identified interactions. In particular, reciprocal co‐immunoprecipitation, proximity‐based assays, endogenous validation strategies, and functional perturbation experiments will be important to establish the biological significance of the identified BICC1‐associated partners.

## 5 Conclusions

This study provides an exploratory characterization of the human BICC1 interactome in HEK293T cells, expanding the current understanding of this understudied RBP. Our analysis reveals a pronounced enrichment of proteins involved in mRNA splicing and components of the PRMT5/WDR77 methylosome complex, thereby identifying previously unrecognized molecular pathways associated with BICC1. Importantly, we demonstrate that the BICC1 interactome is highly enriched in proteins linked to biomolecular condensates, including SGs, P‐bodies, and Cajal bodies, positioning BICC1 as a potential regulator of RNA‐associated membraneless organelles. Although BICC1 localization to SGs has been previously reported [10, 34, 39], our findings extend this observation by implicating BICC1 more broadly in the organization and regulation of biomolecular condensates. This highlights a previously unexplored functional dimension of BICC1 in coordinating RNA–protein assemblies within the cell.

Given the exploratory nature of this study and the limited number of biological replicates, the identified interactions should be interpreted as a discovery‐based resource that will benefit from additional biological and functional validation in future studies. Nevertheless, the interaction framework established here provides a useful foundation for future investigations into the regulatory mechanisms associated with BICC1 function and its contribution to cellular homeostasis. Collectively, these results support a model in which BICC1 acts as an integrative component of phase‐separated RNA–protein networks, with its interactions modulated by RNA and specific protein domains. Given the emerging links between BICC1 dysregulation, cancer, and neurological disorders, further studies will be critical to determine how alterations in its interactome and RNA‐dependent interactions influence disease progression, stress responses, and pathological remodeling of biomolecular condensates.

## Author Contributions

Conceptualization, H.M.A.‐P. and K.B.M.; methodology, H.M.A.‐P., F.H.B., G.A.M., F.E.C., G.O.B., M.H.B., K.B.M.; investigation, H.M.A.‐P. and K.B.M.; resources, H.M.A.‐P., M.H.B. and K.B.M.; writing – original draft preparation, H.M.A.‐P.; writing – review and editing, H.M.A.‐P., C.S., K.B.M.; visualization, H.M.A.‐P.; supervision, M.H.B. and K.B.M.; project administration, H.M.A.‐P. and K.B.M.; funding acquisition, H.M.A.‐P., M.H.B. and K.B.M. All authors have read and agreed to the published version of the manuscript.

## Funding

HMAP was a recipient of a fellowship from Fundação de Amparo à Pesquisa do Estado de São Paulo (FAPESP) (Grant Number: 21/04867‐5) and Conselho Nacional de Desenvolvimento Científico e Tecnológico (CNPq). FHB was a recipient of a fellowship from FAPESP (Grant Number: 2019/11884‐3). KBM was a recipient of an INCT grant (CNPQ 465651/2014‐3) and FAPESP (Grant Number: 20/02006‐0).

## Conflicts of Interest

The authors declare no conflicts of interest.

## Supporting information


**Figure S1:** Evolutionarily conserved features of BICC1 orthologs ( *H. sapiens* , *M. musculus* , *X. laevis* , *D. rerio* , *D. melanogaster* , and *C. elegans* ). The amino acid residues were colored in blue by conversation (> 90%). Each of the KH domains and the associated GXXG motifs (light blue) are indicated, as well as the SAM domain (red boxes). The NCBI database was used for searching Human BICC1 orthologs proteins. The Multiple Sequence Alignment was performed using Clustal Omega that uses seeded guide trees and HMM profile‐profile techniques to generate alignments between three or more sequences. The alignments were generated with Jalview v2.11.2.2 open access software.

**Figure S2:** Confirmation of BICC1 constructs cloning and overexpression in HEK293T cells. (A) BICC1 constructs (FL and ΔSAM) were digested with the restriction enzyme AgeI, resulting in the expected size (8412 and 8220 bp), which only cleaves the BICC1 sequence. Mock (empty vector—pcDNA3.1) was digested with the restriction enzyme EcoRI resulting in the expected size of 5403 bp, as a control of the experiment; (B) Western blot confirming the overexpression of the constructs. Anti‐Flag antibody was used to reveal BICC1 constructs (FL—Full length and ΔSAM—Deletion of SAM domain) and the control GFP. The GAPDH housekeeping gene was used as endogenous control. Each well was loaded with 20 μg of protein sample. M = marker. Supplemental data

**Figure S3:** Determination of RNase A concentration for digestion of RNA using purified RNA samples. M = Marker; lane 1 = 1 ug of RNA not treated with RNase A; lane 2 = 1 ug of RNA treated with 2 ug of RNase A; lane 3 = 1 ug of RNA treated with 5 ug of RNase A; lane 4 = 1 ug of RNA treated with 10 ug of RNase A; lane 5 = 1 ug of RNA treated with 15 ug of RNase A.

**Figure S4:** Technical replicates demonstrate that BICC1 is recruited to stress granules upon sodium arsenite‐induced stress in HEK293 cells through a SAM domain‐dependent process. Confocal microscopy images with a 100× objective (in immersion oil) and 2× zoom in. Cellular stress induction with sodium arsenite (NaAsO2 0.5 mM for 1 h before fixation) causes stress granule maturation with BICC1 recruitment and colocalization with G3BP1 (stress‐granule marker). The SAM domain deletion (ΔSAM) compromises this process. Cell regions were analyzed per replicate to calculate the average Pearson's correlation coefficient, which is highlighted in each experimental condition. Scale bar = 10 μm.

**Figure S5:** Immunofluorescence using the BICC1 antibody and anti‐Flag antibody to detect endogenous and overexpression of BICC in HEK293T. As secondary antibodies, Alexa 555 was used to stain anti‐BICC1, and Alexa 488 was used to stain anti‐Flag. The nucleus was stained with DAPI. Images were obtained using ImageJ software, and all pictures were taken at a magnification of 630X with an Elyra PS.1 Inverted Microscopy (Purple) (super resolution structured illumination). Experiments were performed in triplicate for each condition, and a total of 10 cell regions were analyzed to calculate the average Pearson's correlation coefficient. Scale bars are indicated at 10 μm.

**Figure S6:** Co‐localization of BICC1 and PRMT5. BICC1 was stained with the primary antibody anti‐BICC1, and PRMT5 was stained with the primary antibody anti‐PRMT5. As secondary antibodies, Alexa 555 was used to stain BICC1, and Alexa 488 was used to stain PRMT5. The nucleus was stained with DAPI. Images were obtained using ImageJ open access software, and all pictures were taken at a magnification of 630X with an Elyra PS.1 Inverted Microscopy (Purple) (super‐resolution structured illumination). Experiments were performed in triplicate for each condition, and a total of 10 cell regions were analyzed to calculate the average Pearson's correlation coefficient. Scale bars are indicated at 10 μm.

**Figure S7:** Localization of BICC1 and PARP1. BICC1 was stained with the primary antibody anti‐Flag and PARP1 was stained with the primary antibody anti‐PARP1. As secondary antibodies, Alexa 555 was used to stain BICC1‐Flag, and Alexa 488 was used to stain PARP1. The nucleus was stained with DAPI. Images were obtained using ImageJ open access software and all pictures were taken at a magnification of 630X with an Elyra PS.1 Inverted Microscopy (Purple) (super‐resolution structured illumination). Experiments were performed in triplicate for each condition, and a total of 10 cell regions were analyzed to calculate the average Pearson's correlation coefficient. Scale bars are indicated at 10 μm.

**Figure S8:** Uncropped SDS‐PAGE gels and immunoblotting membranes used in this study. (A) SDS‐PAGE from Figure 1A. (B–D) Immunoblottings from Figure 1B. (E–K) Immnunoblottings from Figure 5A. (L,M) immnunoblottings from Figure 5B. The presence of the co‐eluted antibody light and heavy chains (25 kDa and 50 kDa, respectively) was highlighted. Asterisks colored in red indicate previous membrane staining with another antibody.

**Table S1:** Description of antibodies used in Immunoblotting assays. (PDF).

**Table S2:** Description of antibodies used in immunofluorescence assays. (PDF).

**Table S3:** List of 45 proteins found in the BICC1 full‐length (FL) immunoprecipitation assays in the presence of RNA. (PDF).

**Table S4:** List of 33 proteins found in the BICC1 full‐length (FL) immunoprecipitation assays in the absence of RNA. (PDF).

**Table S5:** List of 51 proteins found in the BICC1 deletion of SAM domain (ΔSAM) immunoprecipitation assays in the presence of RNA. (PDF).

**Table S6:** List of 32 proteins found in the BICC1 deletion of SAM domain ΔSAM immunoprecipitation assays in the absence of RNA. (PDF).

**Table S7:** List of 63 proteins found in the control green fluorescent protein (GFP) immunoprecipitation assays in the presence of RNA. (PDF).

**Table S8:** List of 74 proteins found in the control green fluorescent protein (GFP) immunoprecipitation assays in the absence of RNA. (PDF).

**Table S9:** Proteins identified with RBDs in each experimental condition. (PDF).

**Table S10:** Overlap of the proteins listed in the Database of proteins related to Liquid–Liquid Phase Separation (DrLLPS) with our data of Co‐IP‐MS. (PDF).

**Table S11:** Normalized immunoblotting densitometry values and corresponding normalized LC MS/MS LFQ intensity measurements of validated BICC1 protein partners. (PDF).

**Table S12:** Pearson's correlation obtained from each cell analyzed in the immunofluorescence assays. (PDF).

## Data Availability

DNA sequencing files from cloning constructs are available at Zenodo database: BICC1‐FL https://doi.org/10.5281/zenodo.10251208 and BICC1‐ΔSAM https://doi.org/10.5281/zenodo.10251198. Mass spectrometry raw data have been deposited in the PRIDE repository under accession number PXD054297 and are also available via Zenodo database: https://doi.org/10.5281/zenodo.10443029. Code generated for GO analysis was deposited in the GitHub repository: https://github.com/HeloisaMonteiroAP/Master_BICC1_Project.
