# Supplementary material for: Exploring the BICC1 Interactome in HEK293T Cells: Insights Into RNA‐Mediated Protein Networks and Biomolecular Condensates
Source: FASEB Bioadv. 2026 Aug 7;8(8):e70135. doi: 10.1096/fba.2026-00035 (PMC13450734; doi:10.1096/fba.2026-00035)
Supplement: Supplementary file 1 [file FBA2-8-e70135-s001.pdf]

Table S6. **List of 32 proteins found in the BICC1 deletion of SAM domain ΔSAM immunoprecipitation assays** (in triplicates) in the absence of RNA (+), sorted by their number of unique peptides and average of sequence coverage. (+) Treatment with RNase A.

**UniProtID**	**Gene Name**	**Protein Name**	**#Peptides (1)**	**#Peptides (1)**	**#Peptides (3)**	**Average of Sequence Coverage (%)**	**SP**
**Q9H694**	**BICC1**	**Protein bicaudal C homolog 1**	**37**	**41**	**41**	**44**	**1**
O14744	PRMT5	Protein arginine N-methyltransferase 5	16	16	19	38	**1**
Q9Y3F4	STRAP	Serine-threonine kinase receptor-associated protein	9	12	10	39	**1**
O75534	CSDE1	Cold shock domain-containing protein E1	14	12	15	18	**1**
Q9BQA1	WDR77	Methylosome protein 50	7	7	6	25	**0.99**
P25789	PSMA4	Proteasome subunit alpha type-4	6	7	7	31	**1**
C9JIR6	PPM1B	protein-serine/threonine phosphatase (Fragment)	9	9	8	32	**1**
P27824	CANX	Calnexin	2	1	3	8	0.04
E7ENZ3	CCT5	T-complex protein 1 subunit epsilon	5	1	3	13	0.61
P27348	YWHAQ	14-3-3 protein theta	2	4	5	13	0.34
P63104	YWHAZ	14-3-3 protein zeta/delta	2	3	2	11	**0.85**
Q15208	STK38	Serine/threonine-protein kinase 38	4	6	6	13	**0.98**
E7EX17	EIF4B	Eukaryotic translation initiation factor 4B	4	5	4	10	**0.96**
P40227	CCT6A	T-complex protein 1 subunit zeta	3	2	4	7	**0.94**

P98175	RBM10	RNA-binding protein 10	4	8	5	9	**0.99**
Q15750	TAB1	TGF-beta-activated kinase 1 and MAP3K7-binding protein 1	3	2	3	5	**0.94**
P26641	EEF1G	Elongation factor 1-gamma	3	2	4	0	0.66
A6NMY6	ANXA2	Putative annexin A2-like protein	3	2	0	12	0.41
F8W6I7	HNRNPA1	Heterogeneous nuclear ribonucleoprotein A1	3	3	3	2	**0.72**
P25705	ATP5A1	ATP synthase subunit alpha, mitochondrial	4	1	3	3	**0.8**
Q9NXV2	KCTD5	BTB/POZ domain-containing protein KCTD5	3	2	3	3	**0.91**
A0A7I2V5J8	NPM1	Nucleophosmin	2	3	2	10	0.33
P14678	SNRPB	Small nuclear ribonucleoprotein-associated proteins B and B	2	3	3	0	0.1
P25398	RPS12	40S ribosomal protein S12	2	1	2	0	0.22
P30041	PRDX6	Peroxiredoxin-6	1	3	2	3	**0.78**
P50991	CCT4	T-complex protein 1 subunit delta	5	2	1	15	0.33
P78371	CCT2	T-complex protein 1 subunit beta	5	2	2	0	0.57
A0A499FJE1	BOLA2B	BolA-like protein 2	3	2	2	0	0.15
E9PJD9	RPL27A	60S ribosomal protein L27a	2	1	2	0	0.06

J3KQN4	RPL36A	60S ribosomal protein L36a	3	0	2	5	0.11
P38159	RBMX	RNA-binding motif protein, X chromosome	3	2	4	0	0.26
P49368	CCT3	T-complex protein 1 subunit gamma	4	0	2	3	0.27
Q9NX58	LYAR	Cell growth-regulating nucleolar protein	2	0	2	0	0.16

SP, SAINT probability; SP values greater than 0.7 are highlighted in bold.
