# Supplementary material for: Exploring the BICC1 Interactome in HEK293T Cells: Insights Into RNA‐Mediated Protein Networks and Biomolecular Condensates
Source: FASEB Bioadv. 2026 Aug 7;8(8):e70135. doi: 10.1096/fba.2026-00035 (PMC13450734; doi:10.1096/fba.2026-00035)
Supplement: Supplementary file 2 [file FBA2-8-e70135-s002.pdf]

Figure S1

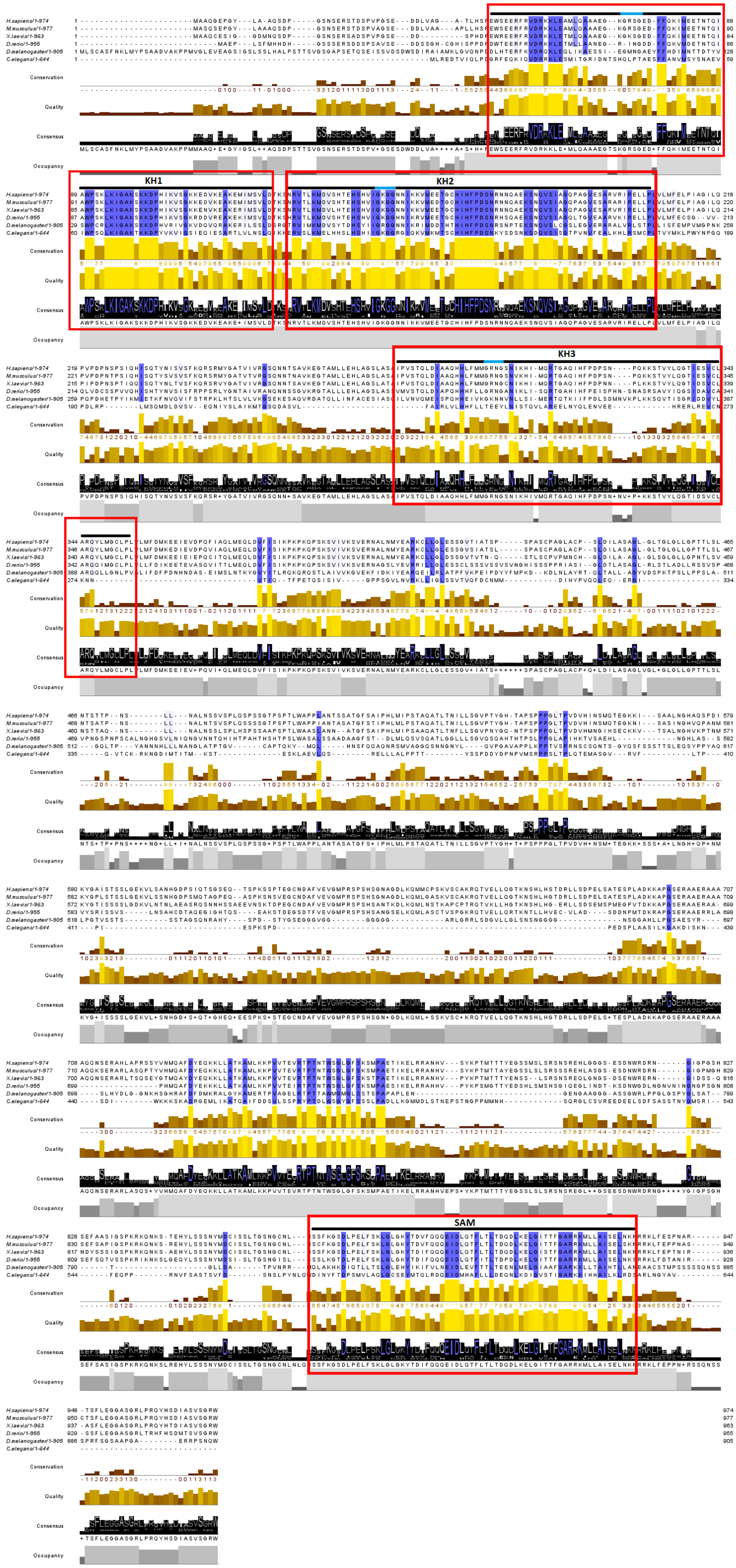

KH1
KH2
KH3
SAM
H.sapiens/1-974
M.musculus/1-977
X.laevis/1-963
D.rerio/1-955
D.melanogaster/1-905
C.elegans/1-644
Conservation
Quality
Consensus
Occupancy
