# Supplementary material for: Exploring the BICC1 Interactome in HEK293T Cells: Insights Into RNA‐Mediated Protein Networks and Biomolecular Condensates
Source: FASEB Bioadv. 2026 Aug 7;8(8):e70135. doi: 10.1096/fba.2026-00035 (PMC13450734; doi:10.1096/fba.2026-00035)
Supplement: Supplementary file 3 [file FBA2-8-e70135-s003.pdf]

Table S12. **Pearson's correlation obtained from each cell analyzed in the immunofluorescence assays.**

		Pearson's correlation
**BICC1 (FL) overexpression (Anti-Flag and Anti-G3BP1) (Without arsenite treatment)**	Cell 1	0.29
	Cell 2	0.20
	Cell 3	0.18
	Cell 4	0.21
	Cell 5	0.08
	Cell 6	0.29
	Cell 7	0.24
	Cell 8	0.03
	Cell 9	0.18
	Cell 10	0.15
	**Average (SD)**	**0.185 (0.07)**
**BICC1 (ΔSAM) overexpression (Anti-Flag and Anti-G3BP1) (Without arsenite treatment)**	Cell 1	0.33
	Cell 2	0.05
	Cell 3	0.31
	Cell 4	0.53
	Cell 5	0.17
	Cell 6	0.65
	Cell 7	0.46
	Cell 8	0.41
	Cell 9	0.28
	Cell 10	0.44
	**Average (SD)**	**0.363 (0.16)**
**BICC1 (FL) overexpression (Anti-Flag and Anti-G3BP1) (With arsenite treatment)**	Cell 1	0.70
	Cell 2	0.78
	Cell 3	0.68
	Cell 4	0.35
	Cell 5	0.50
	Cell 6	0.70
	Cell 7	0.26
	Cell 8	0.60
	Cell 9	0.62

	Cell 10	0.63
	**Average (SD)**	**0.582 (0.15)**
**BICC1 (ΔSAM) overexpression (Anti-Flag and Anti-G3BP1) (With arsenite treatment)**	Cell 1	0.07
	Cell 2	0.07
	Cell 3	0.08
	Cell 4	-0.01
	Cell 5	0.02
	Cell 6	0.38
	Cell 7	0.37
	Cell 8	0.38
	Cell 9	0.39
	Cell 10	0.33
	**Average (SD)**	**0.208 (0.16)**
**BICC1 overexpression (Anti-Flag and anti-BICC1)**	Cell 1	0.84
	Cell 2	0.84
	Cell 3	0.90
	Cell 4	0.92
	Cell 5	0.75
	Cell 6	0.74
	Cell 7	0.54
	Cell 8	0.73
	Cell 9	0.50
	Cell 10	0.73
	**Average (SD)**	**0.749 (0.13)**
**BICC1 overexpression (Anti-BICC1 and anti-PRMT5)**	Cell 1	0.32
	Cell 2	0.42
	Cell 3	0.29
	Cell 4	0.12
	Cell 5	0.26
	Cell 6	0.15
	Cell 7	0.25
	Cell 8	0.33
	Cell 9	0.20

	Cell 10	0.22
	**Average (SD)**	**0.256 (0.08)**
**BICC1 overexpression (Anti-BICC1 and anti-PARP1)**	Cell 1	0.87
	Cell 2	-0.25
	Cell 3	-0.34
	Cell 4	-0.13
	Cell 5	0.20
	Cell 6	0.79
	Cell 7	0.87
	Cell 8	-0.51
	Cell 9	-0.44
	Cell 10	-0.11
	**Average (SD)**	**0.095 (0.55)**
