# Supplementary material for: Exploring the BICC1 Interactome in HEK293T Cells: Insights Into RNA‐Mediated Protein Networks and Biomolecular Condensates
Source: FASEB Bioadv. 2026 Aug 7;8(8):e70135. doi: 10.1096/fba.2026-00035 (PMC13450734; doi:10.1096/fba.2026-00035)
Supplement: Supplementary file 4 [file FBA2-8-e70135-s004.pdf]

Figure S2

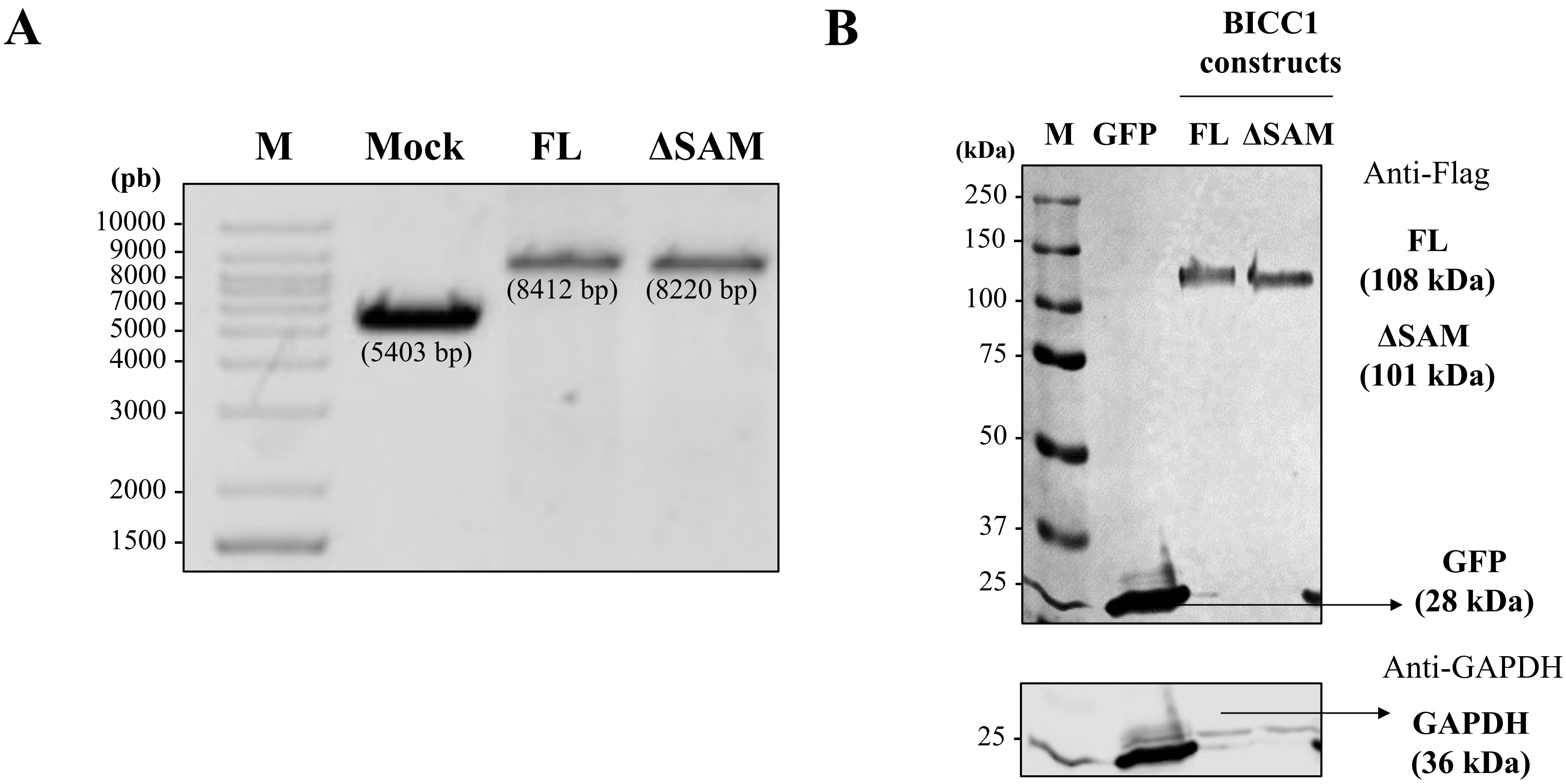
A
M
Mock
FL
ΔSAM
(pb)
10000
9000
8000
7000
5000
4000
3000
2000
1500
(5403 bp)
(8412 bp)
(8220 bp)
B
BICC1 constructs
(kDa)
M
GFP
FL
ΔSAM
Anti-Flag
250
150
100
75
50
37
25
FL (108 kDa)
ΔSAM (101 kDa)
GFP (28 kDa)
Anti-GAPDH
25
GAPDH (36 kDa)
