# Supplementary material for: Exploring the BICC1 Interactome in HEK293T Cells: Insights Into RNA‐Mediated Protein Networks and Biomolecular Condensates
Source: FASEB Bioadv. 2026 Aug 7;8(8):e70135. doi: 10.1096/fba.2026-00035 (PMC13450734; doi:10.1096/fba.2026-00035)
Supplement: Supplementary file 5 [file FBA2-8-e70135-s005.pdf]

Table S2. **Description of antibodies used in immunofluorescence assays**.

Antibody	Host/Isotype	Dilution	Time of incubation	Immunogen	Catalog (Company)
Anti-BICC1 polyclonal antibody	Rabbit	1:100	1 h (RT)	Amino acids 75-107 mapping at the N-terminal region of human BICC1	#PA5-116342 (Thermo Scientific)
Anti-PRMT5 monoclonal antibody	Mouse	1:50	1 h (RT)	Amino acids 338-637 mapping at the C-terminus region of human PRMT5	#sc-376937 (Santa Cruz Biotechnolog y)
Anti-PARP1 monoclonal antibody	Rabbit	1:50	1 h (RT)	Synthetic peptide corresponding to residues surrounding Gly623 of human PARP-1	#9532 (Thermo Scientific)
Anti-G3BP1 polyclonal antibody	Rabbit	1:100	Overnigth (4 ºC)	KLH conjugated synthetic peptide, corresponding to internal region of human G3BP1	#RN048PW (MBL Life science)
Anti-Flag M2 monoclonal antibody	Mouse	1:500	1 h (RT)	Flag peptide sequence: DYKDDDDK	#F1804 (Sigma)
Alexa Fluor™ 488 antibody	Goat anti-Mouse	1:500	1 h (RT)		#A-11001 (Thermo Scientific)
Alexa Fluor™ 555 antibody	Goat anti-Rabbit	1:500	1 h (RT)		#A-21428 (Thermo Scientific)
