# Supplementary material for: Exploring the BICC1 Interactome in HEK293T Cells: Insights Into RNA‐Mediated Protein Networks and Biomolecular Condensates
Source: FASEB Bioadv. 2026 Aug 7;8(8):e70135. doi: 10.1096/fba.2026-00035 (PMC13450734; doi:10.1096/fba.2026-00035)
Supplement: Supplementary file 6 [file FBA2-8-e70135-s006.pdf]

Table S5. **List of 51 proteins found in the BICC1 deletion of SAM domain (ΔSAM) immunoprecipitation assays** (in triplicates) in the presence of RNA (-), sorted by their number of unique peptides and average of sequence coverage. (-) No treatment with RNase A.

**UniProtID**	**Gene Name**	**Protein Name**	**#Peptides (1)**	**#Peptides (1)**	**#Peptides (3)**	**Average of Sequence Coverage (%)**	**SP**
**Q9H694**	**BICC1**	**Protein bicaudal C homolog 1**	**41**	**39**	**39**	**44**	**1**
O14744	PRMT5	Protein arginine N-methyltransferase 5	20	16	24	45	**1**
Q9Y3F4	STRAP	Serine-threonine kinase receptor-associated protein	13	14	12	48	**1**
O75534	CSDE1	Cold shock domain-containing protein E1	26	25	24	30	**1**
Q9BQA1	WDR77	Methylosome protein 50	8	10	11	39	**1**
P25789	PSMA4	Proteasome subunit alpha type-4	4	8	7	31	**0.99**
C9JIR6	PPM1B	Protein-serine/threonine phosphatase (Fragment)	11	11	8	39	**1**
P27824	CANX	Calnexin	3	1	2	15	0.08
E7ENZ3	CCT5	T-complex protein 1 subunit epsilon	4	5	4	11	**0.72**
P27348	YWHAH	14-3-3 protein theta	7	8	8	10	**0.75**
P63104	YWHAZ	14-3-3 protein zeta/delta	5	4	5	23	**0.88**
Q15208	STK38	Serine/threonine-protein kinase 38	9	7	7	19	**1**
A0A0J9YYL3	PUF60	Poly(U)-binding-splicing factor PUF60 (Fragment)	3	5	6	11	0.6
A0A7I2V4N2	NONO	Non-POU domain-containing octamer-binding protein	1	3	3	6	**0.83**
E7EX17	EIF4B	Eukaryotic translation initiation factor 4B	4	5	3	10	**0.97**
P40227	CCT6A	T-complex protein 1 subunit zeta	4	2	6	9	0.69
P62258	YWHAE	14-3-3 protein epsilon	7	3	4	21	**0.82**
P62280	RPS11	40S ribosomal protein S11	4	3	5	20	0.58
P98175	RBM10	RNA-binding protein 10	2	4	6	7	**0.93**

Q15750	TAB1	TGF-beta-activated kinase 1 and MAP3K7-binding protein 1	3	2	4	6	**0.93**
Q9Y295	DRG1	Developmentally-regulated GTP-binding protein 1	0	4	4	9	0.36
A0A8I5KWT8	MYH9	Myosin-9	14	26	0	19	0.66
K7EIY1	FAM32A	Protein FAM32A	2	2	3	4	0.06
P23246	SFPQ	Splicing factor, proline- and glutamine-rich	2	2	4	14	0.56
P26368	U2AF2	Splicing factor U2AF 65 kDa subunit	0	2	3	34	0.06
P26641	EEF1G	Elongation factor 1-gamma	3	2	4	27	0.36
P42677	RPS27	40S ribosomal protein S27	3	3	2	8	0.24
P55072	VCP	Transitional endoplasmic reticulum ATPase	0	3	2	39	0.56
Q04917	YWHAH	14-3-3 protein eta	2	1	3	20	0.56
A0A0G2JLI4	DDX5	Probable ATP-dependent RNA helicase DDX5	2	4	4	3	0.23
A0A2R8YCW1	DDX3X	RNA helicase	1	2	3	6	0.54
F8W6I7	HNRNPA1	Heterogeneous nuclear ribonucleoprotein A1	2	1	2	6	**0.77**
H0YGW7	ABCF1	ATP-binding cassette sub-family F member 1 (Fragment)	1	2	3	0	0.2
O60825	PFKFB2	6-phosphofructo-2-kinase)/fructose-2,6-bisphosphaase 2	2	3	2	4	**0.9**
P25705	ATP5A1	ATP synthase subunit alpha, mitochondrial	5	1	3	5	0.37
P31946	YWHAB	14-3-3 protein beta/alpha	2	2	2	10	0.61
Q9NXV2	KCTD5	BTB/POZ domain-containing protein KCTD5	3	6	3	3	**0.97**
Q9Y237	PIN4	Peptidyl-prolyl cis-trans isomerase NIMA-interacting 4	1	2	2	26	0.01
A0A7I2V3E1	PARP1	Poly [ADP-ribose] polymerase	2	4	1	4	**0.79**

D6RF44	HNRNPD	Heterogeneous nuclear ribonucleoprotein D0 (Fragment)	2	2	1	1	0.52
E7EQV3	PABPC1	Polyadenylate-binding protein	3	3	1	0	**0.82**
F8VPF3	MYL6	Myosin light polypeptide 6 (Fragment)	4	4	0	22	0.6
J3QRS3	MYL12A	Myosin regulatory light chain 12A	3	5	0	23	0.6
O43809	NUDT21	Cleavage and polyadenylation specificity factor subunit 5	3	3	3	1	0.2
P25398	RPS12	40S ribosomal protein S12	3	3	3	0	**0.95**
P78371	CCT2	T-complex protein 1 subunit beta	3	2	4	3	0.43
Q07666	KHDRBS1	KH domain-containing, RNA-binding, signal transduction-associated protein 1	1	2	2	17	0.35
E7ETK5	IMPDH2	Inosine-5-monophosphate dehydrogenase	1	2	2	0	**0.74**
G5E972	TMPO	Lamina-associated polypeptide 2, isoforms beta/gamma	2	1	2	0	**0.7**
P35580	MYH10	Myosin-10	4	8	0	2	0.58
Q7Z406	MYH14	Myosin-14	3	3	0	0	0.54
Q9NZI8	IGF2BP1	Insulin-like growth factor 2 mRNA-binding protein 1	2	1	2	0	**0.7**

SP, SAINT probability; SP values greater than 0.7 are highlighted in bold.
