# Supplementary material for: Exploring the BICC1 Interactome in HEK293T Cells: Insights Into RNA‐Mediated Protein Networks and Biomolecular Condensates
Source: FASEB Bioadv. 2026 Aug 7;8(8):e70135. doi: 10.1096/fba.2026-00035 (PMC13450734; doi:10.1096/fba.2026-00035)
Supplement: Supplementary file 7 [file FBA2-8-e70135-s007.pdf]

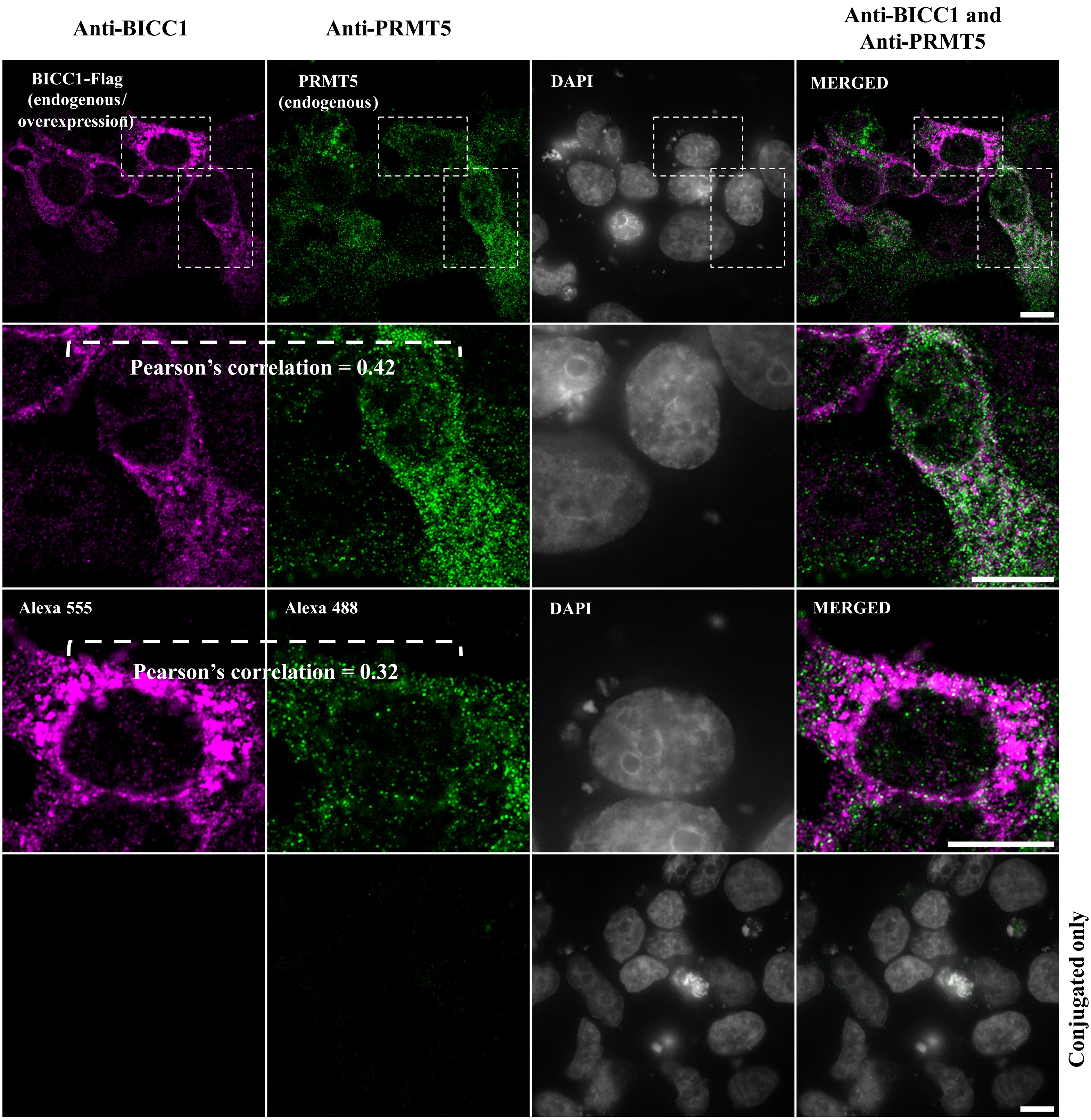
Anti-BICC1
Anti-PRMT5
Anti-BICC1 and Anti-PRMT5
BICC1-Flag (endogenous/ overexpression)
PRMT5 (endogenous)
DAPI
MERGED
Pearson's correlation = 0.42
Alexa 555
Alexa 488
DAPI
MERGED
Pearson's correlation = 0.32
Conjugated only
