# Supplementary material for: Exploring the BICC1 Interactome in HEK293T Cells: Insights Into RNA‐Mediated Protein Networks and Biomolecular Condensates
Source: FASEB Bioadv. 2026 Aug 7;8(8):e70135. doi: 10.1096/fba.2026-00035 (PMC13450734; doi:10.1096/fba.2026-00035)
Supplement: Supplementary file 8 [file FBA2-8-e70135-s008.pdf]

Table S8. **List of 74 proteins found in the control green fluorescent protein (GFP) immunoprecipitation assays** (in triplicates) in the absence of RNA (+), sorted by their number of unique peptides and average of sequence coverage. (+) Treatment with RNase A.

UniProtID	Gene Name	Protein Name	#Peptides (1)	#Peptides (2)	#Peptides (3)	Average of Sequence Coverage (%)
P62701	RPS4X	40S ribosomal protein S4, X isoform	15	13	13	32
H7BY10	RPL23A	60S ribosomal protein L23a (Fragment)	12	10	11	5
P68371	TUBB4B	Tubulin beta-4B chain	9	8	13	28
A0A7I2V659	EEF1A1	Elongation factor 1-alpha	9	8	7	25
P11021	HSPA5	Endoplasmic reticulum chaperone BiP	8	12	13	21
P07437	TUBB	Tubulin beta chain	8	7	13	26
A0A7I2V2G2	HSPA9	Stress-70 protein, mitochondrial	8	10	10	20
P68363	TUBA1B	Tubulin alpha-1B chain	8	8	7	22
P60709	ACTB	Actin, cytoplasmic 1	7	5	6	20
P04259	KRT6B	Keratin, type II cytoskeletal 6B	7	10	8	14
Q06830	PRDX1	Peroxiredoxin-1	7	6	6	35
P39019	RPS19	40S ribosomal protein S19	7	6	5	31
P61247	RPS3A	40S ribosomal protein S3a	7	7	5	0
A0A1W2PPS1	HNRNPU	Heterogeneous nuclear ribonucleoprotein U	6	8	10	12
P62277	RPS13	40S ribosomal protein S13	6	5	5	13
A0A0G2JIW1	HSPA1A	Heat shock 70 kDa protein 1B	5	9	10	16
P62851	RPS25	40S ribosomal protein S25	5	4	3	23
Q86V81	ALYREF	THO complex subunit 4	5	5	5	26
P60866	RPS20	40S ribosomal protein S20	5	4	5	29
P30050	RPL12	60S ribosomal protein L12	5	4	4	0
P10412	H1-4	Histone H1.4	5	4	4	4
A0A8I5KVB5	GTF2I	General transcription factor II-I	4	7	9	8

P05141	SLC25A5	ADP/ATP translocase 2	4	5	3	14
P62269	RPS18	40S ribosomal protein S18	4	2	4	21
P62263	RPS14	40S ribosomal protein S14	4	3	2	3
P63173	RPL38	60S ribosomal protein L38	4	3	2	44
A2NJV5	IGKV2-29	Immunoglobulin kappa variable 2-29	4	3	4	0
H3BNC9	RS17	40S ribosomal protein S17	4	4	4	11
P62316	SNRPD2	Small nuclear ribonucleoprotein Sm D2	4	3	3	46
P62979	RPS27A	Ubiquitin-40S ribosomal protein S27a	4	4	3	3
P08238	HSP90AB1	Heat shock protein HSP 90-beta	4	3	3	5
F8VU65	RPLP0	60S acidic ribosomal protein P0 (Fragment)	4	2	3	0
P11142	HSPA8	Heat shock cognate 71 kDa protein	3	6	8	11
P17066	HSPA6	Heat shock 70 kDa protein 6	3	6	7	10
P12236	SLC25A6	ADP/ATP translocase 3	3	3	1	3
P23396	RPS3	40S ribosomal protein S3	3	6	2	15
P62829	RPL23	60S ribosomal protein L23	3	2	3	25
A0A7P0Z497	PPIB	Peptidyl-prolyl cis-trans isomerase	3	3	5	20
P46783	RPS10	40S ribosomal protein S10	3	5	2	25
P62318	SNRPD3	Small nuclear ribonucleoprotein Sm D3	3	3	3	28
P19474	TRIM21	E3 ubiquitin-protein ligase TRIM21	3	1	2	5
P62273	RPS29	40S ribosomal protein S29	3	3	3	0
A0A075B6R9	IGKV2D-24	Probable non-functional immunoglobulin kappa variable 2D-24	3	2	2	13
P62899	RPL31	60S ribosomal protein L31	3	5	7	28
B5MDF5	RAN	GTP-binding nuclear protein Ran	3	2	1	11
E9PR30	FAU	FAU ubiquitin-like and ribosomal protein S30	3	3	3	0
P12273	PIP	Prolactin-inducible protein	3	2	0	0
Q9BRT6	LLPH	Protein LLP homolog	3	2	2	0
P61978	HNRNPK	Heterogeneous nuclear ribonucleoprotein K	2	1	3	5

P62081	RPS7	40S ribosomal protein S7	2	3	4	25
I3L3P7	RPS15A	40S ribosomal protein S15a	2	2	2	0
P07900	HSP90AA1	Heat shock protein HSP 90-alpha	2	2	3	3
F6VRR5	POLDIP3	Polymerase delta-interacting protein 3	2	2	4	1
P35268	RPL22	60S ribosomal protein L22	2	3	2	0
P32119	PRDX2	Peroxiredoxin-2	2	1	2	4
P61204	ARF3	ADP-ribosylation factor 3	2	2	0	0
A0A7I2V599	HSPD1	60 kDa heat shock protein, mitochondrial	2	3	3	30
P84090	ERH	Enhancer of rudimentary homolog	2	3	3	33
A0A087X2D0	SRSF3	Serine/arginine-rich-splicing factor 3	2	3	3	0
P49411	TUFM	Elongation factor Tu, mitochondrial	2	1	2	0
P04908	H2AC8	Histone H2A type 1-B/E	2	2	2	0
D6RAN4	RPL9	60S ribosomal protein L9 (Fragment)	2	3	2	0
E5RIT6	RPL26L1	60S ribosomal protein L26-like 1 (Fragment)	2	0	2	0
E7ETK0	RPS24	40S ribosomal protein S24	2	2	2	1
P05109	S100A8	Protein S100-A8	2	2	0	11
P62266	RPS23	40S ribosomal protein S23	2	2	1	0
Q92522	H1-10	Histone H1.10	2	2	2	0
Q8NC51	SERBP1	Plasminogen activator inhibitor 1 RNA-binding protein	1	3	2	7
P47813	EIF1AX	Eukaryotic translation initiation factor 1A, X-chromosomal	1	3	3	1
Q14011	CIRBP	Cold-inducible RNA-binding protein	1	2	3	34
P31943	HNRNPH1	Heterogeneous nuclear ribonucleoprotein H	1	2	2	9
P42766	RPL35	60S ribosomal protein L35	1	2	2	0
P50990	CCT8	T-complex protein 1 subunit theta	0	2	2	3
A0A286YF22	PHGDH	D-3-phosphoglycerate dehydrogenase	0	2	2	0
