# Supplementary material for: Exploring the BICC1 Interactome in HEK293T Cells: Insights Into RNA‐Mediated Protein Networks and Biomolecular Condensates
Source: FASEB Bioadv. 2026 Aug 7;8(8):e70135. doi: 10.1096/fba.2026-00035 (PMC13450734; doi:10.1096/fba.2026-00035)
Supplement: Supplementary file 9 [file FBA2-8-e70135-s009.pdf]

Table S11. **Normalized immunoblotting densitometry values and corresponding normalized LC-MS/MS LFQ intensity measurements of validated BICC1 protein partners.**

**Co-IP Conditions**	**Immunoblotting**	**LC-MS/MS**
PRMT5:IP FL(-)	0.287714845	0.27124944
PRMT5:IP FL (+)	0.160874174	0.240516032
PRMT5: IP SAM (-)	0.390016843	0.26599945
PRMT5: IP SAM (+)	0.296184988	0.2370992
PRMT5: IP GFP (-)	0.012089499	0.000001
PRMT5: IP GFP (+)	0.023510097	0.000001

IGF2BP1:IP FL(-)	0.059741174	0.000001
IGF2BP1:IP FL (+)	0.011056403	0.000001
IGF2BP1: IP SAM (-)	0.369205681	0.003303841
IGF2BP1: IP SAM (+)	0.008861982	0.000001
IGF2BP1: IP GFP (-)	0.026427549	0.000001
IGF2BP1: IP GFP (+)	0.017747192	0.000001

PARP1:IP FL(-)	0.026855439	0.000001
PARP1:IP FL (+)	0.013307267	0.000001
PARP1: IP SAM (-)	0.095869352	0.002550114
PARP1: IP SAM (+)	0.011861206	0.000001
PARP1: IP GFP (-)	0.008805644	0.000001
PARP1: IP GFP (+)	0.001484214	0.000001

STK38:IP FL(-)	0.193772628	0.023696904
STK38:IP FL (+)	0.081690434	0.019899339
STK38:SAM (-)	0.35945933	0.025591528
STK38:IP SAM (+)	0.335928107	0.020188595
STK38:IP GFP (-)	0.004329563	0.000001
STK38:IP GFP (+)	0.003822107	0.000001
