# Supplementary material for: Exploring the BICC1 Interactome in HEK293T Cells: Insights Into RNA‐Mediated Protein Networks and Biomolecular Condensates
Source: FASEB Bioadv. 2026 Aug 7;8(8):e70135. doi: 10.1096/fba.2026-00035 (PMC13450734; doi:10.1096/fba.2026-00035)
Supplement: Supplementary file 10 [file FBA2-8-e70135-s010.pdf]

Table S4. **List of 33 proteins found in the BICC1 full-length (FL) immunoprecipitation assays** (in triplicates) in the absence of RNA (+), sorted by their number of unique peptides and average of sequence coverage. (+) Treatment with RNase A.

| **UniProtID** | **Gene Name** | **Protein Name** | **#Peptides (1)** | **#Peptides (1)** | **#Peptides (3)** | **Average of Sequence Coverage (%)** | **SP** |
|---|---|---|---|---|---|---|---|
| **Q9H694** | **BICC1** | **Protein bicaudal C homolog 1** | **41** | **37** | **40** | **42** | **1** |
| O14744 | PRMT5 | Protein arginine N-methyltransferase 5 | 15 | 10 | 14 | 24 | **1** |
| Q9Y3F4 | STRAP | Serine-threonine kinase receptor-associated protein | 3 | 3 | 4 | 12 | **0.96** |
| O75534 | CSDE1 | Cold shock domain-containing protein E1 | 6 | 3 | 6 | 6 | **0.99** |
| Q9BQA1 | WDR77 | Methylosome protein 50 | 7 | 5 | 6 | 23 | **1** |
| P25789 | PSMA4 | Proteasome subunit alpha type-4 | 5 | 5 | 5 | 18 | **0.99** |
| C9JIR6 | PPM1B | Protein-serine/threonine phosphatase (Fragment) | 9 | 6 | 9 | 29 | **1** |
| P27824 | CANX | Calnexin | 2 | 0 | 2 | 6 | 0.03 |
| E7ENZ3 | CCT5 | T-complex protein 1 subunit epsilon | 5 | 2 | 5 | 7 | **0.78** |
| P27348 | YWHAQ | 14-3-3 protein theta | 3 | 2 | 2 | 15 | 0.2 |
| P63104 | YWHAZ | 14-3-3 protein zeta/delta | 2 | 2 | 2 | 10 | **0.87** |
| Q15208 | STK38 | Serine/threonine-protein kinase 38 | 8 | 3 | 5 | 12 | **0.98** |
| E7EX17 | EIF4B | Eukaryotic translation initiation factor 4B | 5 | 3 | 3 | 8 | **0.97** |
| P40227 | CCT6A | T-complex protein 1 subunit zeta | 2 | 1 | 5 | 5 | **0.79** |
| P98175 | RBM10 | RNA-binding protein 10 | 6 | 2 | 6 | 7 | **0.96** |
| Q15750 | TAB1 | TGF-beta-activated kinase 1 and MAP3K7-binding protein 1 | 3 | 1 | 4 | 5 | **0.84** |
| P26641 | EEF1G | Elongation factor 1-gamma | 3 | 0 | 2 | 0 | 0.43 |
| P55072 | VCP | Transitional endoplasmic reticulum ATPase | 4 | 1 | 2 | 0 | **0.84** |
| Q99832 | CCT7 | T-complex protein 1 subunit eta | 4 | 1 | 5 | 6 | 0.69 |
| A0A0G2JLI4 | DDX5 | Probable ATP-dependent RNA helicase DDX5 | 4 | 1 | 2 | 0 | **0.86** |
| F8W6I7 | HNRNPA1 | Heterogeneous nuclear ribonucleoprotein A1 | 3 | 3 | 3 | 2 | **0.77** |

| | | | | | | | |
|---|---|---|---|---|---|---|---|
| P25705 | ATP5A1 | ATP synthase subunit alpha, mitochondrial | 4 | 2 | 4 | 3 | **0.95** |
| Q9NXV2 | KCTD5 | BTB/POZ domain-containing protein KCTD5 | 2 | 2 | 2 | 2 | **0.9** |
| Q9Y237 | PIN4 | Peptidyl-prolyl cis-trans isomerase NIMA-interacting 4 | 2 | 2 | 2 | 10 | 0.4 |
| P14678 | SNRPB | Small nuclear ribonucleoprotein-associated proteins B and B | 2 | 2 | 2 | 0 | 0.04 |
| P30041 | PRDX6 | Peroxiredoxin-6 | 3 | 3 | 3 | 5 | **0.96** |
| P50991 | CCT4 | T-complex protein 1 subunit delta | 3 | 2 | 1 | 8 | 0.34 |
| B1AMW7 | CDC7 | Cell division cycle 7-related protein kinase (Fragment) | 2 | 0 | 2 | 0 | 0.55 |
| C9JXB8 | RPL24 | 60S ribosomal protein L24 | 1 | 2 | 2 | 0 | 0.34 |
| E7ETK5 | IMPDH2 | Inosine-5-monophosphate dehydrogenase | 2 | 0 | 2 | 0 | 0.55 |
| E9PJD9 | RPL27A | 60S ribosomal protein L27a | 2 | 2 | 2 | 2 | 0.17 |
| J3KQN4 | RPL36A | 60S ribosomal protein L36a | 3 | 2 | 0 | 2 | 0.16 |
| P38159 | RBMX | RNA-binding motif protein, X chromosome | 3 | 1 | 4 | 0 | 0.33 |
| X6R700 | CHTOP | Chromatin target of PRMT1 protein | 1 | 3 | 2 | 0 | 0.19 |

SP, SAINT probability; SP values greater than 0.7 are highlighted in bold.
