# Supplementary material for: Exploring the BICC1 Interactome in HEK293T Cells: Insights Into RNA‐Mediated Protein Networks and Biomolecular Condensates
Source: FASEB Bioadv. 2026 Aug 7;8(8):e70135. doi: 10.1096/fba.2026-00035 (PMC13450734; doi:10.1096/fba.2026-00035)
Supplement: Supplementary file 11 [file FBA2-8-e70135-s011.pdf]

Table S3. **List of 45 proteins found in the BICC1 full length (FL) immunoprecipitation assays** (in triplicates) in the presence of RNA (-), sorted by their number of unique peptides and average of sequence coverage. (-) No treatment with RNase A.

**UniProtID**	**Gene Name**	**Protein Name**	**#Peptides (1)**	**#Peptides (1)**	**#Peptides (3)**	**Average of Sequence Coverage (%)**	**SP**
**Q9H694**	**BICC1**	**Protein bicaudal C homolog 1**	**44**	**42**	**39**	**45**	**1**
O14744	PRMT5	Protein arginine N-methyltransferase 5	17	20	17	36	**1**
Q9Y3F4	STRAP	Serine-threonine kinase receptor-associated protein	11	9	11	39	**1**
O75534	CSDE1	Cold shock domain-containing protein E1	10	16	15	17	**1**
Q9BQA1	WDR77	Methylosome protein 50	10	10	10	36	**1**
P25789	PSMA4	Proteasome subunit alpha type-4	8	8	7	35	**1**
C9JIR6	PPM1B	Protein-serine/threonine phosphatase (Fragment)	7	10	10	32	**1**
P27824	CANX	Calnexin	6	2	1	14	0.2
E7ENZ3	CCT5	T-complex protein 1 subunit epsilon	5	5	5	8	**0.82**
P27348	YWHAQ	14-3-3 protein theta	5	8	7	14	0.69
P63104	YWHAZ	14-3-3 protein zeta/delta	5	4	3	18	**0.88**
Q15208	STK38	Serine/threonine-protein kinase 38	5	9	8	18	**1**
A0A0J9YYL3	PUF60	Poly(U)-binding-splicing factor PUF60 (Fragment)	4	5	7	12	**0.75**
A0A7I2V4N2	NONO	Non-POU domain-containing octamer-binding protein	4	4	3	9	**0.95**

E7EX17	EIF4B	Eukaryotic translation initiation factor 4B	4	5	5	10	**0.98**
P40227	CCT6A	T-complex protein 1 subunit zeta	4	3	2	6	0.54
P62258	YWHAE	14-3-3 protein epsilon	4	5	6	22	**0.92**
P62280	RPS11	40S ribosomal protein S11	4	4	4	20	0.6
P98175	RBM10	RNA-binding protein 10	4	7	5	7	**0.99**
Q15750	TAB1	TGF-beta-activated kinase 1 and MAP3K7-binding protein 1	4	3	3	6	**0.96**
Q9Y295	DRG1	Developmentally-regulated GTP-binding protein 1	4	4	4	13	0.61
A0A8I5KWT8	MYH9	Myosin-9	3	13	2	19	**0.9**
K7EIY1	FAM32A	Protein FAM32A	3	1	3	5	0.1
P23246	SFPQ	Splicing factor, proline- and glutamine-rich	3	4	2	14	0.67
P26368	U2AF2	Splicing factor U2AF 65 kDa subunit	3	1	3	31	0.13
P26641	EEF1G	Elongation factor 1-gamma	3	3	3	27	0.41
P42677	RPS27	40S ribosomal protein S27	3	3	3	9	0.3
P55072	VCP	Transitional endoplasmic reticulum ATPase	3	3	3	33	**0.94**
Q04917	YWHAH	14-3-3 protein eta	3	3	2	11	**0.71**
Q99832	CCT7	T-complex protein 1 subunit eta	3	2	3	5	**0.73**
A0A0G2JLI4	DDX5	Probable ATP-dependent RNA helicase DDX5	2	3	4	5	0.2
A0A2R8YCW1	DDX3X	RNA helicase	2	2	4	6	0.68
F8W6I7	HNRNPA1	Heterogeneous nuclear ribonucleoprotein A1	2	3	2	6	**0.9**

H0YGW7	ABCF1	ATP-binding cassette sub-family F member 1 (Fragment)	2	1	3	5	0.19
O60825	PFKFB2	6-phosphofructo-2-kinase/fructose-2,6-bisphosphatase 2	2	2	2	4	**0.86**
P25705	ATP5A1	ATP synthase subunit alpha, mitochondrial	2	3	4	4	0.41
Q15365	PCBP1	Poly(rC)-binding protein 1	2	1	2	20	0.4
Q9NXV2	KCTD5	BTB/POZ domain-containing protein KCTD5	2	4	3	6	**0.94**
Q9Y237	PIN4	Peptidyl-prolyl cis-trans isomerase NIMA-interacting 4	2	2	3	17	0.06
O43809	NUDT21	Cleavage and polyadenylation specificity factor subunit 5	1	2	4	1	0.16
P06576	ATP5B	ATP synthase subunit beta, mitochondrial	1	2	3	0	0.53
P50991	CCT4	T-complex protein 1 subunit delta	1	2	5	10	0.28
P53999	SUB1	Activated RNA polymerase II transcriptional coactivator p15	1	2	2	8	**0.76**
P78371	CCT2	T-complex protein 1 subunit beta	1	2	3	1	0.24
Q8WU90	ZC3H15	Zinc finger CCCH domain-containing protein 15	1	2	2	4	0.79
Q9Y2H1	STK38L	Serine/threonine-protein kinase 38-like	1	3	2	5	**0.76**

SP, SAINT probability; SP values greater than 0.7 are highlighted in bold.
