# Supplementary material for: Exploring the BICC1 Interactome in HEK293T Cells: Insights Into RNA‐Mediated Protein Networks and Biomolecular Condensates
Source: FASEB Bioadv. 2026 Aug 7;8(8):e70135. doi: 10.1096/fba.2026-00035 (PMC13450734; doi:10.1096/fba.2026-00035)
Supplement: Supplementary file 12 [file FBA2-8-e70135-s012.pdf]

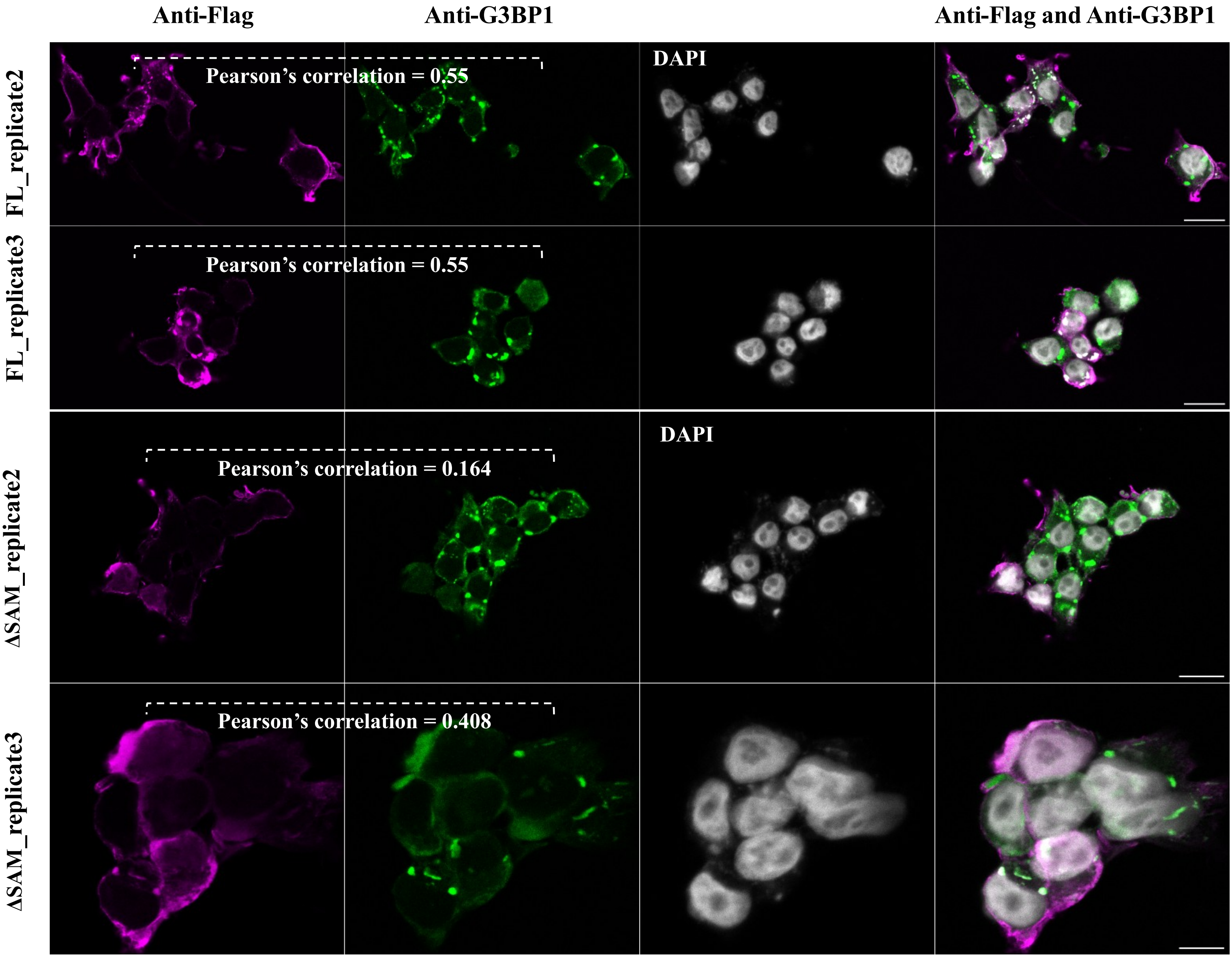

Anti-Flag
Anti-G3BP1
Anti-Flag and Anti-G3BP1
FL_replicate2
Pearson's correlation = 0.55
DAPI
FL_replicate3
Pearson's correlation = 0.55
ΔSAM_replicate2
Pearson's correlation = 0.164
DAPI
ΔSAM_replicate3
Pearson's correlation = 0.408
