# Supplementary material for: Exploring the BICC1 Interactome in HEK293T Cells: Insights Into RNA‐Mediated Protein Networks and Biomolecular Condensates
Source: FASEB Bioadv. 2026 Aug 7;8(8):e70135. doi: 10.1096/fba.2026-00035 (PMC13450734; doi:10.1096/fba.2026-00035)
Supplement: Supplementary file 13 [file FBA2-8-e70135-s013.pdf]

Table S10. **Overlap of the proteins listed in the Database of proteins related to Liquid-Liquid Phase Separation (DrLLPS) with our data of Co-IP-MS.**

UniProt ID	Gene name	Condensate
**Q9H694**	**BICC1**	P-body, Stress granule
P09651	HNRNPA1	Droplet, Nuclear speckle, Nucleolus, Paraspeckle, Sam68 nuclear body, Stress granule, Postsynaptic density, Others
P06748	NPM1	Droplet, Nucleolus, Centrosome/Spindle pole body, Stress granule
O00571	DDX3X	Droplet, Nucleolus, P-body, Stress granule, Postsynaptic density
Q14103	HNRNPD	Droplet, Nucleolus, Sam68 nuclear body, Stress granule
P11940	PABPC1	Nucleolus, P-body, Stress granule, Postsynaptic density
Q15233	NONO	Nuclear speckle, Nucleolus, Paraspeckle, Neuronal granule, P-body, Stress granule
O14744	PRMT5	P-body, Stress granule, Postsynaptic density
Q9Y3F4	STRAP	Gemini of cajal body, Stress granule, Postsynaptic density
O75534	CSDE1	P-body, Stress granule
P55072	VCP	Nucleolus, Stress granule, Postsynaptic density
Q15365	PCBP1	Nuclear speckle, Nucleolus, P-body, Stress granule, Postsynaptic density
Q9NZI8	IGF2BP1	P-body, Stress granule, Others
P23588	EIF4B	Stress granule
Q04917	YWHAH	Stress granule, Postsynaptic density
P17844	DDX5	Nucleolus, Stress granule
P78371	CCT2	Nucleolus, Postsynaptic density
P48643	CCT5	Nucleolus, Postsynaptic density
P25398	RPS12	Nucleolus
Q99832	CCT7	Nucleolus, Postsynaptic density
Q9NX58	LYAR	Nucleolus
P53999	SUB1	Nucleolus
P26368	U2AF2	Nuclear speckle, Nucleolus
P26641	EEF1G	Nucleolus, Stress granule
P40227	CCT6A	Nucleolus, Stress granule, Postsynaptic density
P09874	PARP1	Nucleolus, Centrosome/Spindle pole body, Stress granule, Postsynaptic density
P14678	SNRPB	Nucleolus
P83731	RPL24	Nucleolus, Postsynaptic density
P30041	PRDX6	Stress granule, Postsynaptic density
P62280	RPS11	Nucleolus, Stress granule, Postsynaptic density
P07355	ANXA2	Nucleolus, Postsynaptic density
P23246	SFPQ	Nuclear speckle, Nucleolus, Paraspeckle, Stress granule
P35579	MYH9	Nucleolus, Postsynaptic density
P31946	YWHAB	Stress granule, Postsynaptic density
Q8NE71	ABCF1	Nucleolus, Stress granule
Q15208	STK38	Centrosome/Spindle pole body
P25789	PSMA4	Nucleolus
Q9UHX1	PUF60	P-body
P46776	RPL27A	Nucleolus
P42677	RPS27	Nucleolus, Postsynaptic density
P49368	CCT3	Cajal body, Postsynaptic density

P38159	RBMX	Nucleolus, Sam68 nuclear body, P-body, Stress granule
O43809	NUDT21	Nucleolus
P35580	MYH10	P-body, Postsynaptic density
P62258	YWHAE	Nucleolus, Centrosome/Spindle pole body, Postsynaptic density
P50991	CCT4	Nucleolus, Postsynaptic density
Q07666	KHDRBS1	Nucleolus, Sam68 nuclear body, Stress granule
Q9Y2H1	STK38L	Centrosome/Spindle pole body, Postsynaptic density
P27348	YWHAQ	Stress granule, Postsynaptic density
P60660	MYL6	Postsynaptic density
Q7Z406	MYH14	Postsynaptic density
P63104	YWHAZ	Postsynaptic density
