# Supplementary material for: Exploring the BICC1 Interactome in HEK293T Cells: Insights Into RNA‐Mediated Protein Networks and Biomolecular Condensates
Source: FASEB Bioadv. 2026 Aug 7;8(8):e70135. doi: 10.1096/fba.2026-00035 (PMC13450734; doi:10.1096/fba.2026-00035)
Supplement: Supplementary file 15 [file FBA2-8-e70135-s015.pdf]

Table S1. **Description of antibodies used in western blotting assays.**

Antibody	Host/Isotype	Dilution	Time of incubation	Immunogen	MW (kDa)	Catalog (Company)
Anti-BICC1 polyclonal Antibody	Rabbit	1:1,000	Overnight (4 °C)	Amino acids 75-107 mapping at the N-terminal region of human BICC1	104	#PA5-116342 (Thermo Scientific)
Anti-PRMT5 monoclonal antibody	Rabbit	1:1,000	Overnight (4 °C)	Synthetic peptide. This information is proprietary to Abcam and/or its suppliers	72	#ab109451 (Abcam)
Anti-IGF2BP1 polyclonal antibody	Mouse	1:1,000	Overnight (4 °C)	Recombinant full-length IGF2BP1	63	#RN001M (MBL Life science)
Anti-CCT2 antibody	Rabbit	1:10,000	Overnight (4 °C)	Synthetic peptide. This information is proprietary to Abcam and/or its suppliers.	57	#ab109184 (Abcam)
Anti-PARP1 monoclonal antibody	Rabbit	1:2,000	Overnight (4 °C)	Synthetic peptide corresponding to residues surrounding Gly623 of human PARP-1	116	#9532 (Thermo Scientific)
Anti-HNRNPA1 polyclonal antibody	Rabbit	1:1,000	Overnight (4 °C)	HNRNPA1 fusion protein Ag1656	34	#11176-1-AP (Proteintech)
Anti-PABP polyclonal antibody	Rabbit	1:1,000	Overnight (4 °C)	Synthetic peptide conjugated to KLH derived from within residues 600 to the C-terminus of Human PABP	75	#ab21060 (Abcam)
Anti-GAPDH polyclonal antibody	Mouse	1:10.000	1 h (RT)	GAPDH fusion protein Ag0766	36	#60004-1 (Proteintech)
Anti-GAPDH polyclonal antibody	Rabbit	1:10,000	1 h (RT)	Synthetic peptide corresponding to residues surrounding Lys260 of human GAPDH protein.	37	#2118 (Cell Signaling)
Anti-Flag® M2 monoclonal antibody	Mouse	1:10,000	1 h (RT)	Flag peptide sequence: DYKDDDDK	Diverse	#F1804 (Sigma)
IRDye® 680RD antibody	Goat anti-Mouse	1:10,000	1 h (RT)	-		#926-68070 (LI-COR)
IRDye® 680RD antibody	Goat anti-Rabbit	1:10,000	1 h (RT)	-		#926-68071 (LI-COR)

IRDye® 800CW antibody	Goat anti-Mouse	1:10,000	1 h (RT)	-	#926-32210 (LI-COR)
IRDye® 800CW antibody	Goat anti-Rabbit	1:10,000	1 h (RT)	-	#926-32211 (LI-COR)
