# Supplementary material for: Exploring the BICC1 Interactome in HEK293T Cells: Insights Into RNA‐Mediated Protein Networks and Biomolecular Condensates
Source: FASEB Bioadv. 2026 Aug 7;8(8):e70135. doi: 10.1096/fba.2026-00035 (PMC13450734; doi:10.1096/fba.2026-00035)
Supplement: Supplementary file 17 [file FBA2-8-e70135-s017.pdf]

Table S9. **Proteins identified with RBDs in each experimental condition**.

Experimental conditions	KH domain	RRM domain	DDX domain	CSD domain	Total number of proteins with RBDs
**FL (-)** (RNA/SAM dependent)	**PCBP1**#	PUF60#, NONO#, EIF4B, RBM10#, SFPQ#, HNRNPA1#, U2AF2#	DDX3X	CSDE1	**10**
**FL (+)** (SAM dependent)	NA	RBM10#, EIF4B, HNRNPA1#, RBMX	NA	CSDE1	**5**
**ΔSAM (-)** (RNA dependent and SAM independent)	**IGF2BP1***, **KHDRBS1**#	EIF4B, PUF60#, SFPQ#, RBM10#, **PABPC1**#, **HNRNPD**, HNRNPA1#, NONO# U2AF2#	DDX3X	CSDE1	**13**
**ΔSAM (+)** (RNA/SAM independent)	NA	EIF4B, RBM10#, HNRNPA1#, RBMX#	NA	CSDE1	**5**

NA = Not appeared; "*" = IGF2BP1 has also RRM domain. "#" = splicing-related proteins. **Highlighted proteins** = Proteins that are exclusive of each experimental condition according to the filtering steps that were determined. (-) No treatment with RNase A; (+) Treatment with RNase A.
