# Supplementary material for: Exploring the BICC1 Interactome in HEK293T Cells: Insights Into RNA‐Mediated Protein Networks and Biomolecular Condensates
Source: FASEB Bioadv. 2026 Aug 7;8(8):e70135. doi: 10.1096/fba.2026-00035 (PMC13450734; doi:10.1096/fba.2026-00035)
Supplement: Supplementary file 18 [file FBA2-8-e70135-s018.pdf]

Figure S8

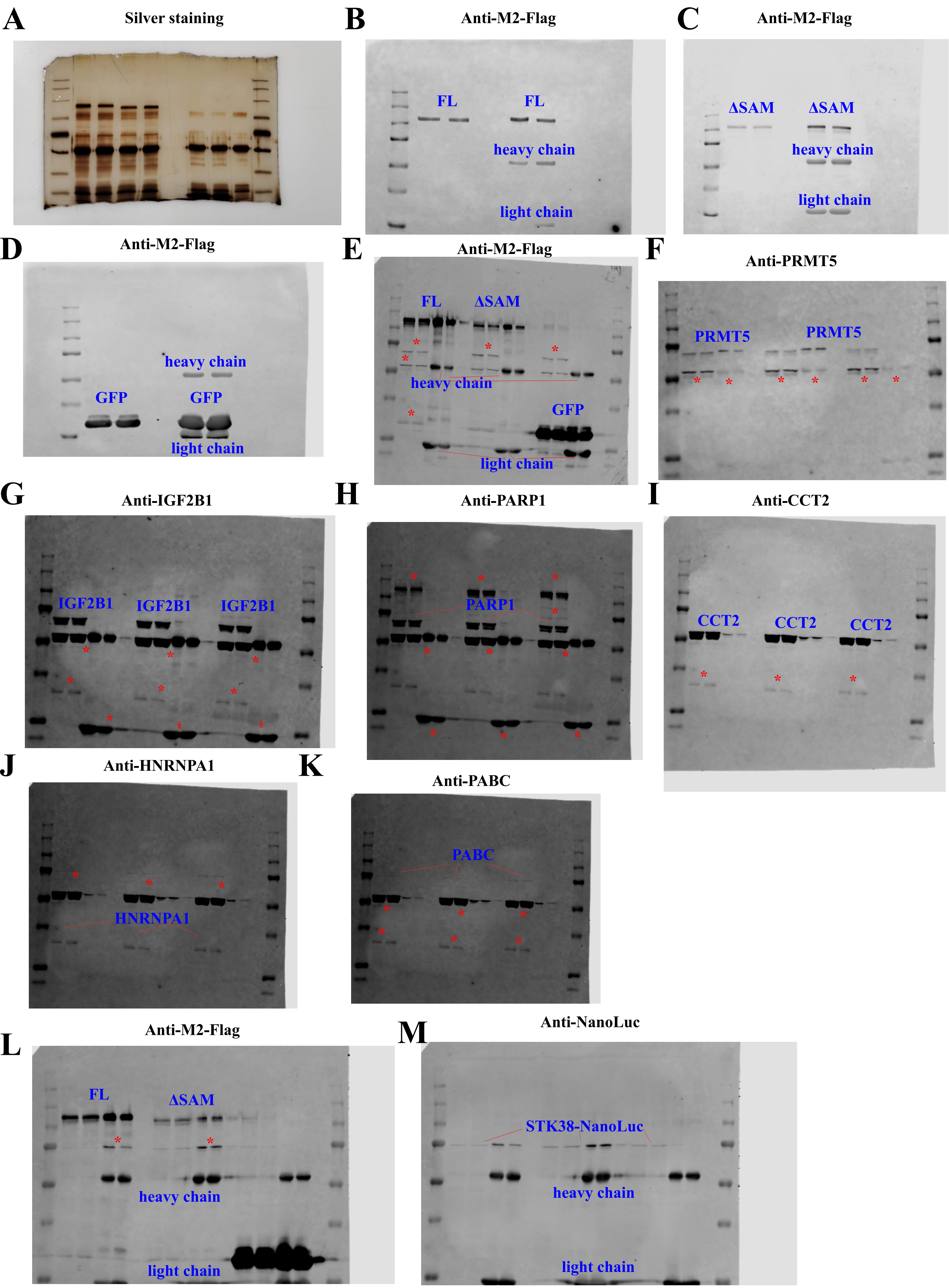
A
Silver staining
B
Anti-M2-Flag
FL
FL
heavy chain
light chain
C
Anti-M2-Flag
ΔSAM
ΔSAM
heavy chain
light chain
D
Anti-M2-Flag
heavy chain
GFP
GFP
light chain
E
Anti-M2-Flag
FL
ΔSAM
heavy chain
GFP
light chain
F
Anti-PRMT5
PRMT5
PRMT5
G
Anti-IGF2B1
IGF2B1
IGF2B1
IGF2B1
H
Anti-PARP1
PARP1
I
Anti-CCT2
CCT2
CCT2
CCT2
J
Anti-HNRNPA1
HNRNPA1
K
Anti-PABC
PABC
L
Anti-M2-Flag
FL
ΔSAM
heavy chain
light chain
M
Anti-NanoLuc
STK38-NanoLuc
heavy chain
light chain
