# Supplementary material for: Exploring the BICC1 Interactome in HEK293T Cells: Insights Into RNA‐Mediated Protein Networks and Biomolecular Condensates
Source: FASEB Bioadv. 2026 Aug 7;8(8):e70135. doi: 10.1096/fba.2026-00035 (PMC13450734; doi:10.1096/fba.2026-00035)
Supplement: Supplementary file 19 [file FBA2-8-e70135-s019.pdf]

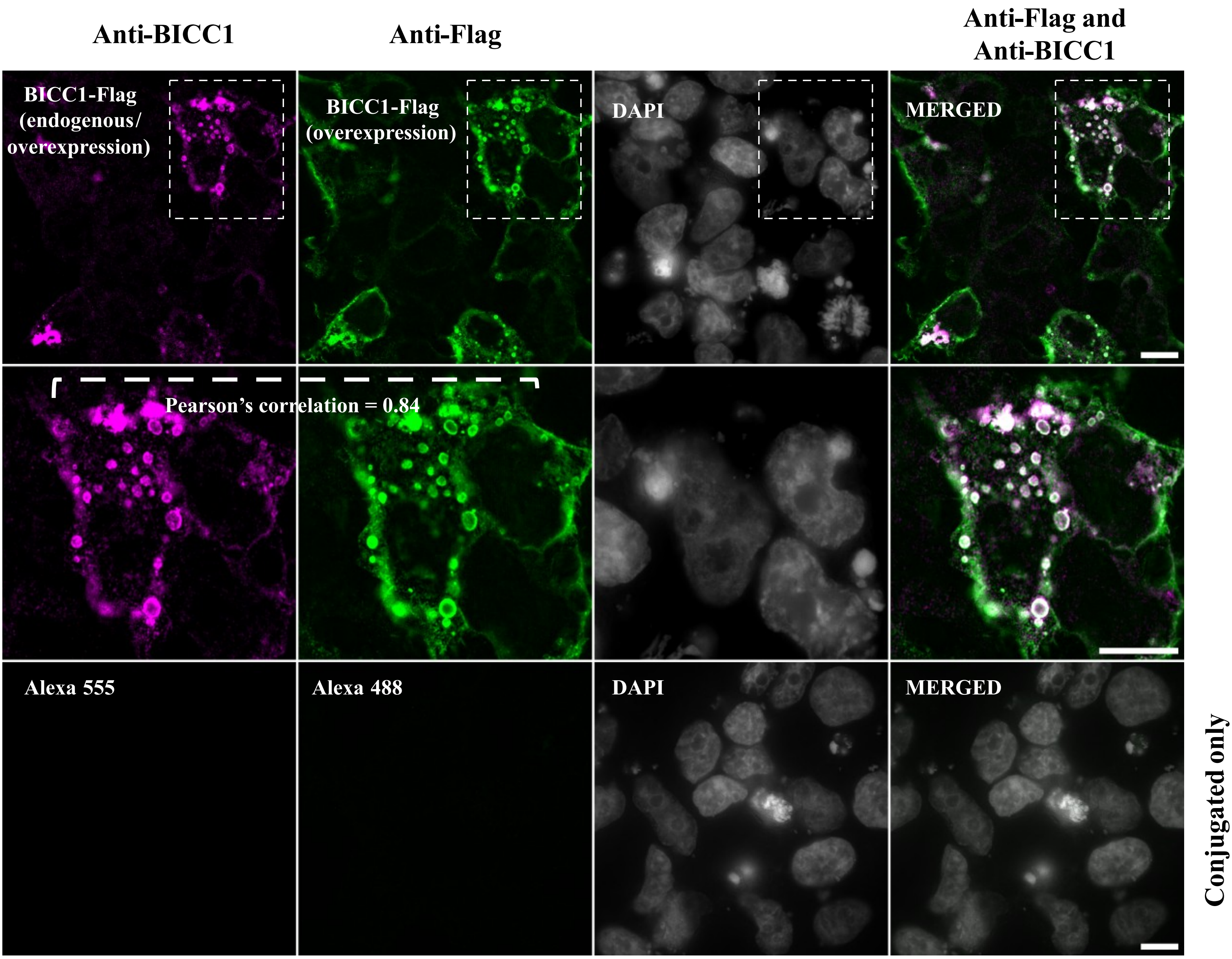
Anti-BICC1
Anti-Flag
Anti-Flag and Anti-BICC1
BICC1-Flag (endogenous/ overexpression)
BICC1-Flag (overexpression)
DAPI
MERGED
Pearson's correlation = 0.84
Alexa 555
Alexa 488
DAPI
MERGED
Conjugated only
