# Supplementary material for: Exploring the BICC1 Interactome in HEK293T Cells: Insights Into RNA‐Mediated Protein Networks and Biomolecular Condensates
Source: FASEB Bioadv. 2026 Aug 7;8(8):e70135. doi: 10.1096/fba.2026-00035 (PMC13450734; doi:10.1096/fba.2026-00035)
Supplement: Supplementary file 20 [file FBA2-8-e70135-s020.pdf]

Table S7. **List of 63 proteins found in the control green fluorescent protein (GFP) immunoprecipitation assays** (in triplicates) in the presence of RNA (-), sorted by their number of unique peptides and average of sequence coverage. (-) No treatment with RNase A.

| UniProtID | Gene Name | Protein Name | #Peptides (1) | #Peptides (2) | #Peptides (3) | Average of Sequence Coverage (%) |
|---|---|---|---|---|---|---|
| A0A8I5KVB5 | GTF2I | General transcription factor II-I | 19 | 18 | 15 | 18 |
| A0A0G2JIW1 | HSPA1A | Heat shock 70 kDa protein 1B | 17 | 20 | 20 | 35 |
| P11021 | HSPA5 | Endoplasmic reticulum chaperone BiP | 16 | 13 | 12 | 26 |
| P11142 | HSPA8 | Heat shock cognate 71 kDa protein | 15 | 16 | 17 | 28 |
| P68371 | TUBB4B | Tubulin beta-4B chain | 12 | 12 | 12 | 37 |
| A0A7I2V659 | EEF1A1 | Elongation factor 1-alpha | 11 | 11 | 10 | 34 |
| P07437 | TUBB | Tubulin beta chain | 10 | 11 | 12 | 34 |
| A0A7I2V2G2 | HSPA9 | Stress-70 protein, mitochondrial | 9 | 7 | 11 | 20 |
| P05141 | SLC25A5 | ADP/ATP translocase 2 | 8 | 7 | 7 | 25 |
| P17066 | HSPA6 | Heat shock 70 kDa protein 6 | 8 | 8 | 8 | 13 |
| P62249 | RPS16 | 40S ribosomal protein S16 | 8 | 5 | 6 | 45 |
| P12236 | SLC25A6 | ADP/ATP translocase 3 | 8 | 6 | 6 | 3 |
| P04259 | KRT6B | Keratin, type II cytoskeletal 6B | 7 | 11 | 9 | 13 |
| P62269 | RPS18 | 40S ribosomal protein S18 | 7 | 8 | 10 | 44 |
| P23396 | RPS3 | 40S ribosomal protein S3 | 7 | 9 | 8 | 36 |
| P68363 | TUBA1B | Tubulin alpha-1B chain | 6 | 9 | 13 | 28 |
| Q06830 | PRDX1 | Peroxiredoxin-1 | 6 | 4 | 6 | 30 |
| P62263 | RPS14 | 40S ribosomal protein S14 | 6 | 6 | 6 | 15 |
| P62829 | RPL23 | 60S ribosomal protein L23 | 6 | 5 | 5 | 49 |

| | | | | | | |
|---|---|---|---|---|---|---|
| A0A7P0Z497 | PPIB | Peptidyl-prolyl cis-trans isomerase | 5 | 9 | 8 | 37 |
| P39019 | RPS19 | 40S ribosomal protein S19 | 5 | 6 | 5 | 29 |
| P46783 | RPS10 | 40S ribosomal protein S10 | 5 | 5 | 5 | 33 |
| P63173 | RPL38 | 60S ribosomal protein L38 | 5 | 5 | 5 | 50 |
| P62851 | RPS25 | 40S ribosomal protein S25 | 5 | 5 | 5 | 30 |
| P61978 | HNRNPK | Heterogeneous nuclear ribonucleoprotein K | 4 | 3 | 5 | 11 |
| Q8NC51 | SERBP1 | Plasminogen activator inhibitor 1 RNA-binding protein | 4 | 5 | 6 | 16 |
| P62081 | RPS7 | 40S ribosomal protein S7 | 4 | 2 | 2 | 20 |
| Q14331 | FRG1 | Protein FRG1 | 4 | 3 | 5 | 19 |
| A2NJV5 | IGKV2-29 | Immunoglobulin kappa variable 2-29 | 4 | 3 | 4 | 0 |
| P47813 | EIF1AX | Eukaryotic translation initiation factor 1A, X-chromosomal | 4 | 4 | 4 | 8 |
| P62318 | SNRPD3 | Small nuclear ribonucleoprotein Sm D3 | 4 | 4 | 4 | 0 |
| Q86V81 | ALYREF | THO complex subunit 4 | 4 | 2 | 3 | 15 |
| P60866 | RPS20 | 40S ribosomal protein S20 | 4 | 3 | 3 | 26 |
| Q14011 | CIRBP | Cold-inducible RNA-binding protein | 4 | 4 | 4 | 0 |
| P35637 | FUS | RNA-binding protein FUS | 3 | 3 | 3 | 5 |
| A0A0A0MR02 | VDAC2 | Voltage-dependent anion-selective channel protein 2 (Fragment) | 3 | 1 | 2 | 13 |
| H3BNC9 | RS17 | 40S ribosomal protein S17 | 3 | 4 | 3 | 9 |
| P19474 | TRIM21 | E3 ubiquitin-protein ligase TRIM21 | 3 | 3 | 3 | 7 |
| P62273 | RPS29 | 40S ribosomal protein S29 | 3 | 3 | 3 | 1 |
| P62701 | RPS4X | 40S ribosomal protein S4, X isoform | 3 | 3 | 5 | 48 |

| | | | | | | |
|---|---|---|---|---|---|---|
| C9JYQ9 | RPL22L1 | 60S ribosomal protein L22-like 1 | 3 | 3 | 2 | 0 |
| P07900 | HSP90AA1 | Heat shock protein HSP 90-alpha | 2 | 2 | 3 | 3 |
| F6VRR5 | POLDIP3 | Polymerase delta-interacting protein 3 | 2 | 3 | 3 | 1 |
| P48047 | ATP5PO | ATP synthase subunit O, mitochondrial | 2 | 3 | 2 | 5 |
| Q9Y383 | LUC7L2 | Putative RNA-binding protein Luc7-like 2 | 2 | 2 | 5 | 10 |
| A0A075B6R9 | IGKV2D-24 | Probable non-functional immunoglobulin kappa variable 2D-24 | 2 | 2 | 3 | 13 |
| P36542 | ATP5C1 | ATP synthase subunit gamma, mitochondrial | 2 | 1 | 4 | 4 |
| Q5T7C4 | HMGB1 | High mobility group protein B1 | 2 | 2 | 3 | 5 |
| Q96CT7 | CCDC124 | Coiled-coil domain-containing protein 124 | 2 | 1 | 5 | 13 |
| B0QYK0 | EWSR1 | RNA-binding protein EWS | 2 | 2 | 1 | 3 |
| P35268 | RPL22 | 60S ribosomal protein L22 | 2 | 3 | 2 | 1 |
| P62913 | RPL11 | 60S ribosomal protein L11 | 2 | 2 | 2 | 0 |
| P62979 | RPS27A | Ubiquitin-40S ribosomal protein S27a | 2 | 2 | 2 | 13 |
| P32119 | PRDX2 | Peroxiredoxin-2 | 2 | 1 | 2 | 0 |
| Q07021 | C1QBP | Complement component 1 Q subcomponent-binding protein, mitochondrial | 2 | 2 | 1 | 0 |
| P08238 | HSP90AB1 | Heat shock protein HSP 90-beta | 1 | 2 | 3 | 3 |
| A0A7I2V599 | HSPD1 | 60 kDa heat shock protein, mitochondrial | 1 | 2 | 3 | 38 |
| Q96AG4 | LRRC59 | Leucine-rich repeat-containing protein 59 | 1 | 2 | 3 | 8 |
| P49411 | TUFM | Elongation factor Tu, mitochondrial | 1 | 2 | 2 | 14 |
| Q14498 | RBM39 | RNA-binding protein 39 | 1 | 4 | 2 | 1 |
| B5MDF5 | RAN | GTP-binding nuclear protein Ran | 1 | 2 | 3 | 11 |

| | | | | | | |
|---|---|---|---|---|---|---|
| P98179 | RBM3 | RNA-binding protein 3 | 1 | 2 | 2 | 0 |
| P12273 | PIP | Prolactin-inducible protein | 1 | 2 | 2 | 0 |
